# Integrating single-cell RNA sequencing with spatial transcriptomics reveal the fibrosis-related genes in hepatocellular carcinoma

**DOI:** 10.3389/fimmu.2025.1659404

**Published:** 2026-01-14

**Authors:** Wenying Qiao, Lei Li, Ronghua Jin, Caixia Hu

**Affiliations:** 1 Interventional Therapy Center for Oncology, Beijing You’an Hospital, Capital Medical University, Beijing, China; 2 Beijing Key Laboratory of Emerging Infectious Diseases, Institute of Infectious Diseases, Beijing Ditan Hospital, Capital Medical University, Beijing, China; 3 Beijing Institute of Infectious Disease, Beijing Ditan Hospital, Capital Medical University, Beijing, China; 4 National Key Laboratory of Intelligent Tracking and Forecasting for Infectious Diseases, Beijing Ditan Hospital, Capital Medical University, Beijing, China

**Keywords:** hepatocellular carcinoma (HCC), liver fibrosis, prognostic gene, single-cell RNA sequencing (scRNA-seq), spatial transcriptomics

## Abstract

**Background:**

Hepatocellular carcinoma (HCC) remains a leading cause of cancer-related mortality, with limited efficacy of current therapies in advanced cases. As a key risk factor for HCC, liver fibrosis may influence tumor progression and immune responses. However, fibrosis-related therapeutic targets remain poorly defined. This study aimed to identify fibrosis-related genes in HCC tumor microenvironment (TME).

**Methods:**

Our research integrated single-cell RNA sequencing (GSE149614), spatial transcriptomics (GSE245908), and bulk RNA-seq data to identify fibrosis-related prognostic genes in HCC. The genes were selected via the Random Survival Forest algorithm. Additionally, bioinformatics analyses were conducted to explore gene expression patterns, immune infiltration, and spatial localization. Key genes were further validated through in EDU incorporation assay, Transwell migration assay, and CCK-8 proliferation assay.

**Results:**

Firstly, single-cell analysis identified endothelial cells as key fibrosis-associated cluster in HCC. Three fibrosis-related prognostic genes, LUC7L3, CREB1, and YIPF4, were further identified and validated to patient survival, immune infiltration, and metabolic activity. In addition, enrichment and drug sensitivity analyses linked key genes to tumor-related pathways and chemotherapy response. Spatial transcriptomics then confirmed the spatial distribution and interactions of these genes. Lastly, cellular assays showed that YIPF4 promoted proliferation and migration of HCC cells.

**Conclusion:**

In this study, we identified fibrosis-related prognostic genes in HCC, including LUC7L3, CREB1, and YIPF4. The roles of these genes in TME were further explored through relevant analyses, potentially providing clinical evidence to support decision-making in HCC management.

## 1 Introduction

Hepatocellular carcinoma (HCC), the most prevalent type of primary liver cancer, is the sixth most prevalent cancer type and the third leading cause of cancer-related mortality (1, 2). Despite the increasing diversity of radical therapeutic strategies for HCC, including surgical resection, local ablation, and liver transplantation, their effectiveness remains constrained, particularly in advanced or recurrent cases (3, 4). While targeted therapies and immunotherapies have demonstrated promising outcomes in HCC patients during these years, their efficacy is observed in only approximately 30% of cases, with a median overall survival of less than two years (5). One potential reason is the dysfunction and impairment of immune cells in the tumor microenvironment (TME), which composed of cancer cells, immune cells, and other components, with its heterogeneity having a substantial impact on patient prognosis (6). Therefore, identifying novel biomarkers and reliable therapeutic targets based on the TME of HCC remains a current research focus.

As a pathological process characterized by excessive extracellular matrix deposition resulting from chronic liver injury, liver fibrosis is a progressive condition that could eventually advance to cirrhosis, liver failure, and HCC (7–9). During HCC therapy, the effect of immune checkpoint inhibitors is subject to individual variability, and factors such as liver fibrosis may influence therapeutic outcomes (10–13). A major challenge is the lack of specific fibrosis-related therapeutic targets, which limits the effectiveness of current treatment strategies aimed at improving the prognosis of patients. Although numerous studies have consistently highlighted the role of immunotherapy in the treatment of HCC, the underlying mechanisms by which fibrosis affects immune regulation and tumor progression remain to be fully elucidated (14, 15). Thus, investigating significant genes associated with HCC, particularly those implicated in fibrosis, may facilitate the identification of novel therapeutic targets and contribute to the optimization of immunotherapy strategies for HCC. Increasing evidence suggests that endothelial cells (ECs) play a critical regulatory role in liver fibrosis. While fibroblasts are widely recognized as the main effector cells responsible for extracellular matrix deposition, recent studies have demonstrated that dysfunctional ECs act as key upstream drivers of fibrogenesis by promoting endothelial-to-mesenchymal transition, secreting pro-fibrotic cytokines, and modulating immune cell infiltration (16–18). Through these mechanisms, ECs indirectly activate hepatic stellate cells and amplify fibrotic remodeling, highlighting their central role in shaping the fibrotic tumor microenvironment.

Single-cell RNA sequencing (scRNA-seq) provides detailed transcriptomic information for individual cells, enabling comparisons between cell clusters and offering deeper insights into cellular heterogeneity (19). This technology not only helps to provide a comprehensive gene expression landscape but also uncovers cellular heterogeneity and its relevance to tumor progression (20). Spatial transcriptomics, as an extension of single-cell sequencing, adds spatial context to gene expression data by mapping gene activity onto tissue sections (21). This method enables the localization of specific gene expression within tissue sections and exhibits the roles of cell subpopulations interacting across different regions (22, 23). By integrating spatial transcriptomics with scRNA-seq, our study offered a comprehensive view of the cellular landscape of HCC and the microenvironment, contributing to advances in precision medicine.

In summary, liver fibrosis serves as a pivotal pathological foundation for HCC, and effective treatment, such as immunotherapy, remains a considerable clinical challenge. Although previous studies have proposed prognostic gene signatures for HCC, research specifically focusing on fibrosis-related genes and their mechanistic roles in HCC progression is still limited. Given the established importance of fibrosis as a key risk factor and its capacity to shape an immunosuppressive tumor microenvironment, identifying fibrosis-specific molecular drivers is essential for advancing therapeutic strategies. Therefore, by integrating single-cell RNA sequencing, spatial transcriptomics, and bulk cohorts, this study aimed to identify fibrosis-related prognostic genes in HCC and evaluate their functional and clinical significance.

## 2 Methods

### 2.1 Data acquisition

The Gene Expression Omnibus (GEO) database (https://www.ncbi.nlm.nih.gov/geo/info/datasets.html), maintained by the National Center for Biotechnology Information (NCBI), serves as a comprehensive repository for gene expression data. We retrieved scRNA-seq data from the GEO dataset GSE149614 (24), which includes samples from 10 HCC patients, consisting of paired tumor and adjacent non-tumor tissues. Additionally, we acquired the GSE245908 (25) spatial transcriptome data file to download 2 samples with complete spatial transcriptome expression profiles for spatial transcriptome analysis.

The Cancer Genome Atlas (TCGA) database (https://portal.gdc.cancer.gov/) is the largest cancer gene information database, storing data such as gene expression, copy number variation, and single nucleotide polymorphism. We downloaded the raw mRNA expression data from the HCC data.

### 2.2 Single-cell data quality control

The expression profiles were processed using the “Seurat” (v4.3.0) package (26). Initial filtering of cells was based on criteria including the total number of unique molecular identifiers (UMI) per cell, the number of genes expressed, and the proportion of mitochondrial gene expression. Cells with a high proportion of mitochondrial gene expression (>10%) were removed as they typically indicate dying cells. The median absolute deviation (MAD) method was employed for outlier detection, and cells with values exceeding 3 MAD from the median were excluded. Doublet cells were identified and filtered using “DoubletFinder” (v2.0.4) (27).

### 2.3 Single-cell data dimensionality reduction clustering and cell annotation

We used the global normalization method to adjust the total expression of each cell to 10,000 by multiplying a coefficient s0, followed by logarithmic transformation. Cell cycle scores were calculated using the “CellCycleScoring” function. We also applied the “FindVariableFeatures” function to identify highly variable genes and normalized gene expression using “ScaleData” to standardize the dataset. Furthermore, Linear dimensionality reduction on the expression matrix and the selection of principal components were performed using “RunPCA”. Harmony (v0.1.1) (28) was then used to remove the batch effect, RunUMAP was used to generate the UMAP embedding for nonlinear dimensionality reduction and visualization. By querying CellMarker databases and literature, manual cell annotation was utilized to find the cell types existing in the corresponding tissues and the corresponding marker genes.

### 2.4 Ligand receptor interaction analysis

“CellChat” (v1.1.3) (29) is a useful tool that enables quantitative inference and analysis of cell-to-cell communication networks from single-cell data. It uses network analysis and pattern recognition methods to predict the main signal inputs and outputs of cells, and how these cells and signals coordinate function. In our study, we used the normalized single-cell expression profile as input data, with the cell subtypes derived from the single-cell analysis serving as the cell-related information. We analyzed the cell-related interactions and quantified the intensity and count of cell-to-cell interactions to observe the activity and impact of each cell type in the disease.

### 2.5 Identify significant genes

We performed feature selection using the “randomForestSRC” (v3.1.0) (30) software package. To identify fibrosis-related genes, we applied the Random Survival Forest algorithm (NREP = 1000, indicating 1000 iterations in Monte Carlo simulations). Genes with a relative importance score >0.1 were selected as significant markers. This selection threshold was empirically determined from the importance score distribution in this dataset to ensure model stability and interpretability.

### 2.6 Tumor immune microenvironment analysis

The CIBERSORT (31) method is a widely used method for evaluating immune cell types in the tumor microenvironment, which contains 547 biomarkers and distinguishes 22 human immune cell phenotypes. Based on the principle of support vector regression, the expression matrix of immune cell subtypes was deconvoluted. In this study, the CIBERSORT algorithm was applied to the bulk RNA-seq expression data from the TCGA-LIHC cohort to estimate the relative proportions of 22 immune cell subsets. Only samples with CIBERSORT-derived p < 0.05 were retained for downstream correlation analyses.

### 2.7 Gene set enrichment analysis and gene set variation analysis

According to the expression of key genes, the patients were divided into high- and low- expression groups, and the differences in signaling pathways between two groups were further analyzed by gene set enrichment analysis (GSEA) (32). GSEA is a phenotype-driven method that identifies significantly enriched pathways based on a ranked list of differentially expressed genes between predefined groups. Fibrosis score was calculated using the HALLMARK_EPITHELIAL_MESENCHYMAL_TRANSITION (EMT) gene signature obtained from the MSigDB database. The background gene set was the annotated version 7.0 gene set downloaded from the MsigDB database. Differential expression analysis of the pathway between groups was performed, and significantly enriched gene sets (adjusted p-value < 0.05) were ranked by the consistency score.

In addition, Gene Set Variation Analysis (GSVA) (v1.42.0) (33) was performed to quantify pathway activity at the individual-sample level. Unlike GSEA, which relies on phenotype grouping, GSVA is a non-parametric, unsupervised method that evaluates variations in pathway enrichment across samples independent of group definition. The GSVA algorithm generated enrichment scores for each pathway, enabling comparative functional profiling and visualization of biological heterogeneity among samples.

### 2.8 Drug susceptibility analysis

Based on the largest pharmacogenomics database, which called Cancer Drug Susceptibility Genomics Database (GDSC) (https://www.cancerrxgene.org/), we used the R software package “oncoPredict” (v1.2) (34) to predict the chemosensitivity of each tumor sample. The IC50 values for each drug were estimated through regression analysis, and ten-fold cross-validation was performed to test the regression and prediction accuracy. The oncoPredict algorithm was used to estimate the half-maximal inhibitory concentration (IC50) values of multiple anticancer drugs based on gene expression profiles, using the GDSC and CCLE reference datasets. This analysis served as an exploratory prediction of potential drug response patterns rather than direct experimental validation.

### 2.9 Dimensionality reduction and clustering of spatial transcriptome sequencing data

Spatial transcriptome data were processed using the “Seurat” package. Raw UMI counts were normalized via regularized negative binomial regression as part of the package “SCTransform”. PCA principal component analysis was used to perform linear dimension reduction for the top 3000 genes with the largest change in expression level, and Uniform Manifold Approximation and Projection (UMAP) algorithm was used for nonlinear dimensionality reduction. Finally, clustering was performed using the Louvain algorithm.

### 2.10 Deconvolution

Robust cell-type decomposition (RCTD) (v1.2) (35) is a supervised learning method that breaks down an RNA sequencing mixture into individual cell types, enabling the assignment of cell types to spatial transcriptomic pixels. In this study, RCTD was applied to the spatial transcriptomics dataset (GSE245908), using the annotated scRNA-seq dataset GSE149614 as the reference to construct cell type–specific expression profiles and guide spatial cell-type mapping.

A related challenge in supervised cell type learning is the platform effect, which refers to the effect of technology-dependent library preparation on the capture rate of individual genes between sequencing platforms. These effects have previously been identified in comparisons of single-cell RNA-seq and single-nucleated RNA-seq (snRNA-seq) on the same biological sample, but RCTD addresses these plateau effects and can be applied to deconvolution analysis of the spatial transcriptome across different platforms. It can accurately discover the localization of cell types in simulated and real-world spatial transcriptome data. RCTD was run with default settings, and the confidence of cell type assignment was assessed using the rho score. Most spatial spots exhibited high-confidence mapping (rho > 0.8), and the resulting spatial distribution patterns were consistent with the single-cell reference annotations.

### 2.11 Space cell interactions and spatial trajectory inference

MISTy (36) facilitates a deeper understanding of marker interactions by analyzing intracellular and intercellular relationships. Its component modeling approach allows for a flexible definition of spatial perspectives, capturing cell type-specific interactions and pathway activities across anatomical regions. Each view of MISTy is considered as a potential source of variability in measuring labeled expressions, and the contribution of each view to the overall expression of each marker is then analyzed. The measured contribution highlights the relevance of potential interaction sources from different spatial environments and is estimated using view-specific models.

Additionally, we employed the STlearn module, a Python library designed for spatial transcriptome data analysis. It provides a range of capabilities for tasks such as data preprocessing, spatial clustering, differential representation analysis, and visualization. By leveraging STlearn’s preprocessing capabilities, we normalized the raw spatial transcriptome data to eliminate the impact of technical variation. Subsequently, we identified different cell populations and analyzed their spatial distribution through the Louvain clustering algorithm and the Leiden clustering algorithm.

### 2.12 EDU incorporation assay

Cell proliferation was evaluated using an EDU incorporation assay. Briefly, cells were incubated with 10 μM EDU for 2 hours at 37°C, followed by fixation in 4% paraformaldehyde and permeabilization with 0.5% Triton X-100. Cells were subsequently stained with Apollo567 dye and counterstained with DAPI. EdU-positive cells were detected using a flow cytometer, and the proliferation rate was calculated as the percentage of EdU-positive cells.

### 2.13 Transwell assay

A total of 5 × 10^4^ cells suspended in serum-free medium were seeded into the upper chamber of a Transwell insert. The lower chamber was filled with medium supplemented with 10% fetal bovine serum to serve as a chemoattractant. After 24 hours of incubation at 37°C, non-migrated cells on the upper surface of the membrane were carefully removed, while migrated cells on the lower surface were fixed with 4% paraformaldehyde, stained with crystal violet, and counted under a light microscope.

### 2.14 CCK-8 proliferation assay

Cell proliferation was assessed using the Cell Counting Kit-8. Cells were seeded into 96-well plates at a density of 2 × 10³ cells per well. At the indicated time points, 10 μL of CCK-8 solution was added to each well and incubated for 2 hours at 37°C. The absorbance was then measured at 450 nm using a microplate reader. Cell viability was expressed as the relative absorbance compared to the control group.

### 2.15 Statistical analysis

All statistical analyses were conducted using R software (version 4.1.3). For comparisons between two groups, continuous variables were assessed using either the independent t-test or the Mann–Whitney U test. Categorical variables were compared using the chi-square test or Fisher’s exact test. Correlation analyses were performed using Spearman regression analysis. A P-value less than 0.05 was considered indicative of statistical significance.

## 3 Results

### 3.1 Single-cell RNA-seq database processing

**Figure 1:**
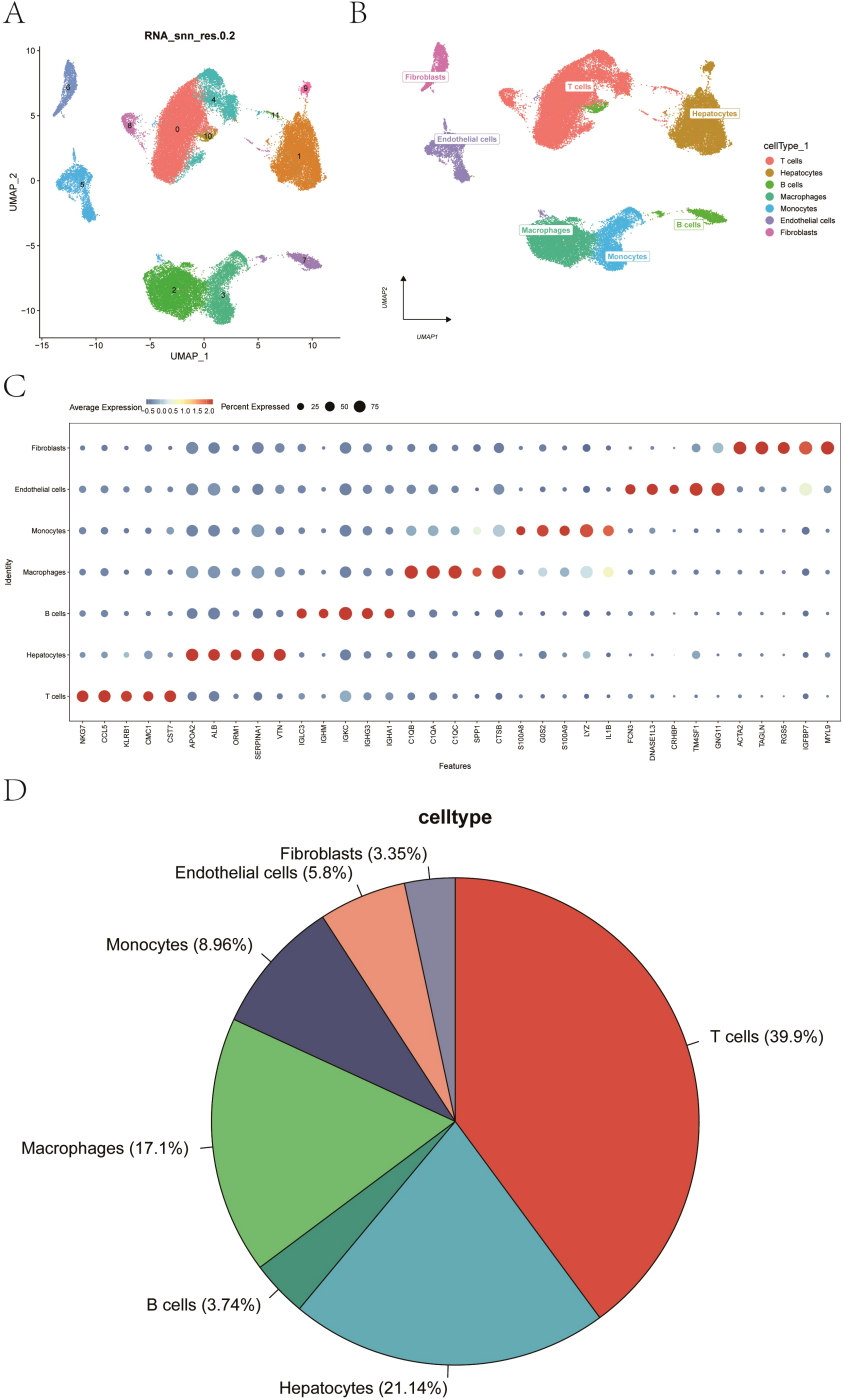
Single-cell RNA-seq analysis reveals liver cell populations. **(A)** UMAP plot after filtering and normalization. **(B)** UMAP visualization highlighting seven major cell types. **(C)** Bubble plot displaying the expression of canonical marker genes across the seven cell clusters. **(D)** Pie chart indicating the proportion of each cell type. *P < 0.05; *** P < 0.001; ****P < 0.0001.

Considering the data quality of multiple samples, low-quality cells were filtered based on threshold criteria, excluding cells with fewer than 200 detected genes, extremely low or high UMI counts, or a high proportion of mitochondrial reads. Cells derived from portal vein tumor thrombus (PVTT) and metastatic lymph node samples were removed, and only primary tumor and non-tumor liver cells were retained for downstream analysis. Subsequently, the double cells were filtered using the package of “DoubletFinder”, with a total of 54,825 cells were retained. The quality control before and after filtration was displayed in **Supplementary Figure S1**. After normalization and dimensionality reduction of the data, we obtained the distribution of the seven cell classes by UMAP visualization, including hepatocytes, B cells, T cells, macrophages, monocytes, endothelial cells, and fibroblasts (**Figures 1A, B**). Cell annotation was performed manually based on the expression of canonical marker genes referenced from the CellMarker databases, combined with published literature and biological knowledge. Representative markers used for cell-type identification included GNG11 and TM4SF1 for endothelial cells, ACTA2 and TAGLN for fibroblasts, ALB and APOA2 for hepatocytes, NKG7 and CCL5 for T cells, IGHM and IGKC for B cells, LYZ and IL1B for monocytes, and C1QA and C1QC for macrophages (**Figure 1C**). Bubble plot of the classic marker of seven clusters and pie chart of the corresponding cell proportions were shown in **Figures 1C, D**, respectively.

**Figure 2:**
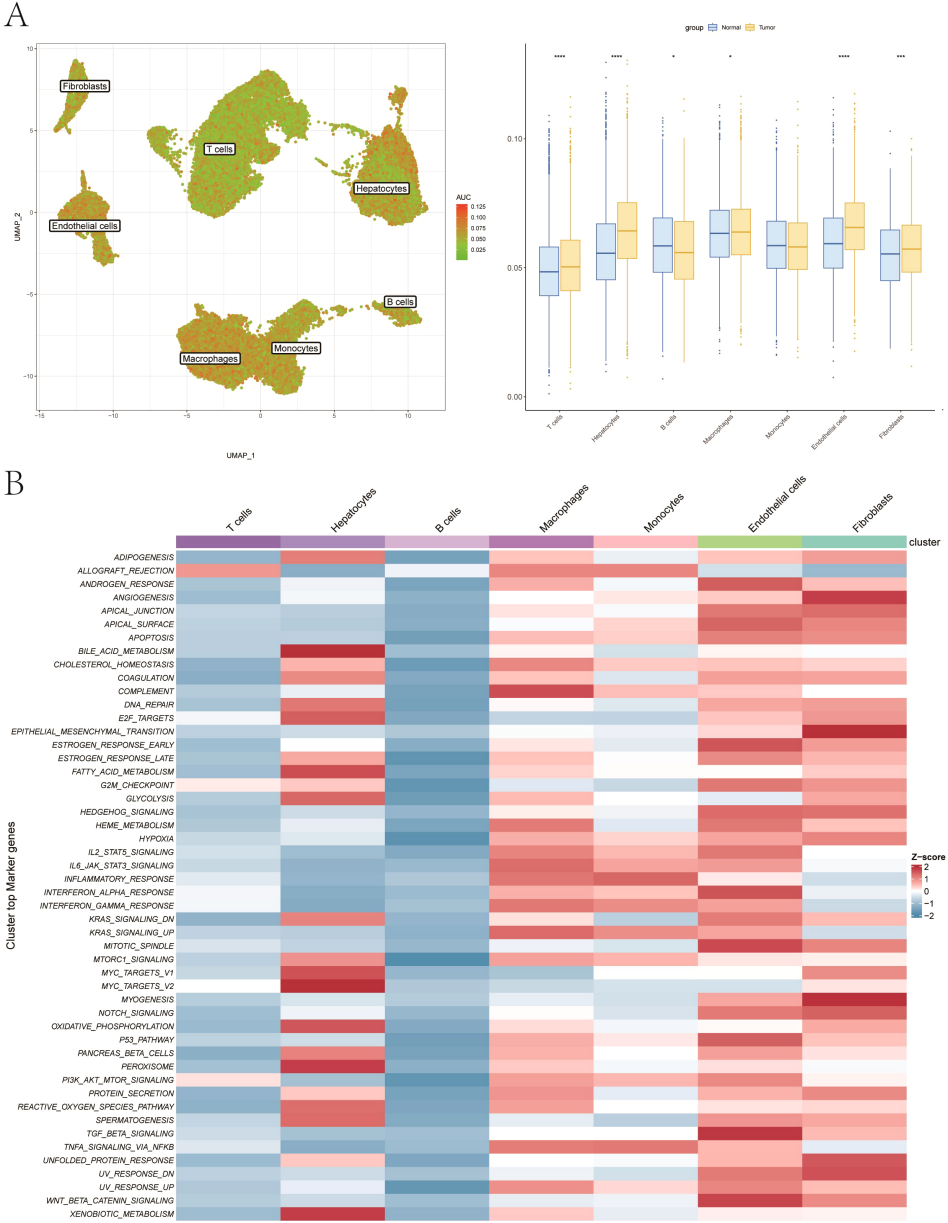
Endothelial cells display highest fibrosis and pathway activity. **(A)** Fibrosis scores by AUCell across cell types. **(B)** Pathway activity heatmap showing elevated signals in endothelial cells.

We further quantified the fibrosis fraction using a gene set obtained from the MSigDB database (https://www.gsea-msigdb.org/gsea/index.jsp), which contained a total of 150 related genes. The “AUCell” algorithm was then utilized to score the activity of gene set at the single-cell level via the fibrosis-related genes. The result revealed significant differences in the cell-derived fibrosis scores between the control and disease groups in most cell groups, with endothelial cells showing the most pronounced difference (**Figure 2A**). Therefore, we selected endothelial cells as the key cell population for subsequent analysis. Furthermore, the HALLMARK gene sets were obtained from the MSigDB database using the “msigdbr” package, and pathway activity in each cell cluster was analyzed using the “AverageHeatmap” function of the “scRNAtoolVis” package. The heat map showed that endothelial cells exhibited relatively high scores across the most of pathways (**Figure 2B**).

### 3.2 Ligand-receptor interaction analysis and enrichment analysis

**Figure 3:**
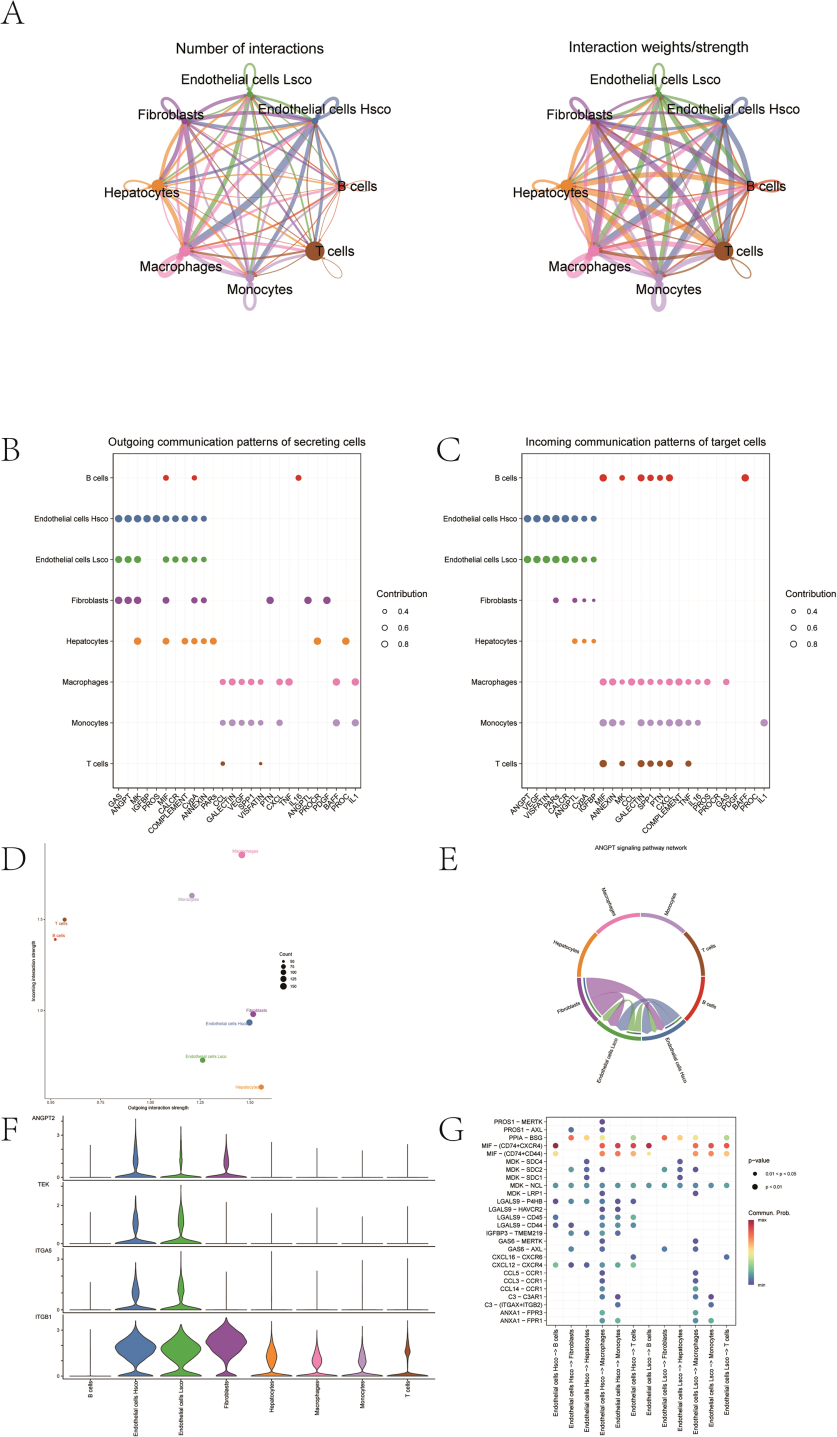
Cell–cell communication of endothelial subgroups. **(A)** Ligand–receptor interactions between high- and low-activity endothelial cells with other cells. **(B, C)** Signal input and output strength across all pathways. **(D–F)** The activity of pathways in endothelial cells, shown by scatter, chord, and violin plots. **(G)** Dot plot showing correlations between endothelial interactions and others.

The classification was based on the median value of the fibrosis-related score computed using the “AddModuleScore” function in Seurat, with cells above and below the median defined as high-activity and low-activity endothelial cells, respectively. We then performed ligand–receptor interaction analysis on the single-cell expression profiles of the two groups based on the “CellChat” package, which revealed complex interaction pairs between these subgroups (**Figure 3A**). Additionally, we calculated the signal reception and transmission intensities of all cells across all signaling pathways (**Figures 3B, C**). Of note, the ANGPT signaling pathway exhibited notably higher reception and transmission intensities in endothelial cells, and was therefore selected as a key pathway for further analysis. As for the reception and transmitting intensity, scatter plot, chord plot, and violin plot under the ANGPT signaling pathway were then plotted (**Figures 3D–F**). Finally, we also created dot plot of the correlations of high-activity endothelial interaction and low-activity endothelial interaction with other cell-to-cell pathways (**Figure 3G**). Among endothelial subclusters, the ANGPT signaling pathway exhibited the strongest incoming and outgoing communication probabilities, consistent with its pro-angiogenic and fibrotic remodeling functions. Moreover, MIF–CD74 signaling was enriched between high-fibrosis endothelial cells and macrophages, suggesting a reciprocal activation that may amplify inflammatory responses and extracellular matrix deposition, thereby linking endothelial signaling to fibrosis and tumor progression.

**Figure 4:**
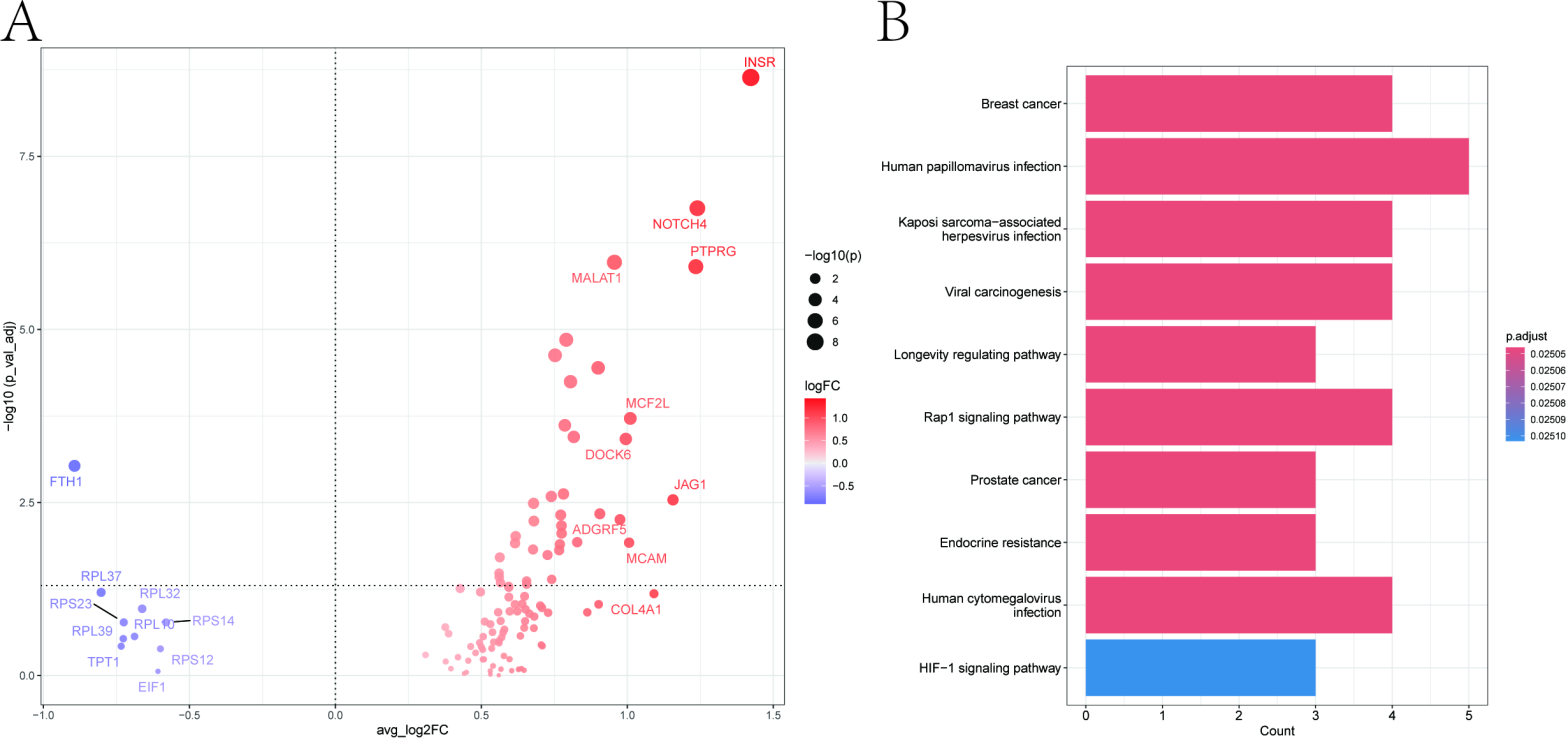
Marker gene analysis of endothelial subgroups. **(A)** Volcano plot of 38 marker genes differentially expressed between endothelial subgroups. **(B)** KEGG enrichment analysis showing top pathways.

After removing genes expressed in less than 10% expressed among cells, we utilized the “FindMarkers” function to identify marker genes of two endothelial cell subgroups. Using a significance threshold of p < 0.05, we selected 38 marker genes, which were visualized in a volcano plot (**Figure 4A**). Furthermore, Kyoto Encyclopedia of Genes and Genomes enrichment analysis based on marker genes showed that the main enrichment pathways included Rap1 signaling pathway and HIF−1 signaling pathway (**Figure 4B**).

### 3.3 Identification of significant genes

**Figure 5:**
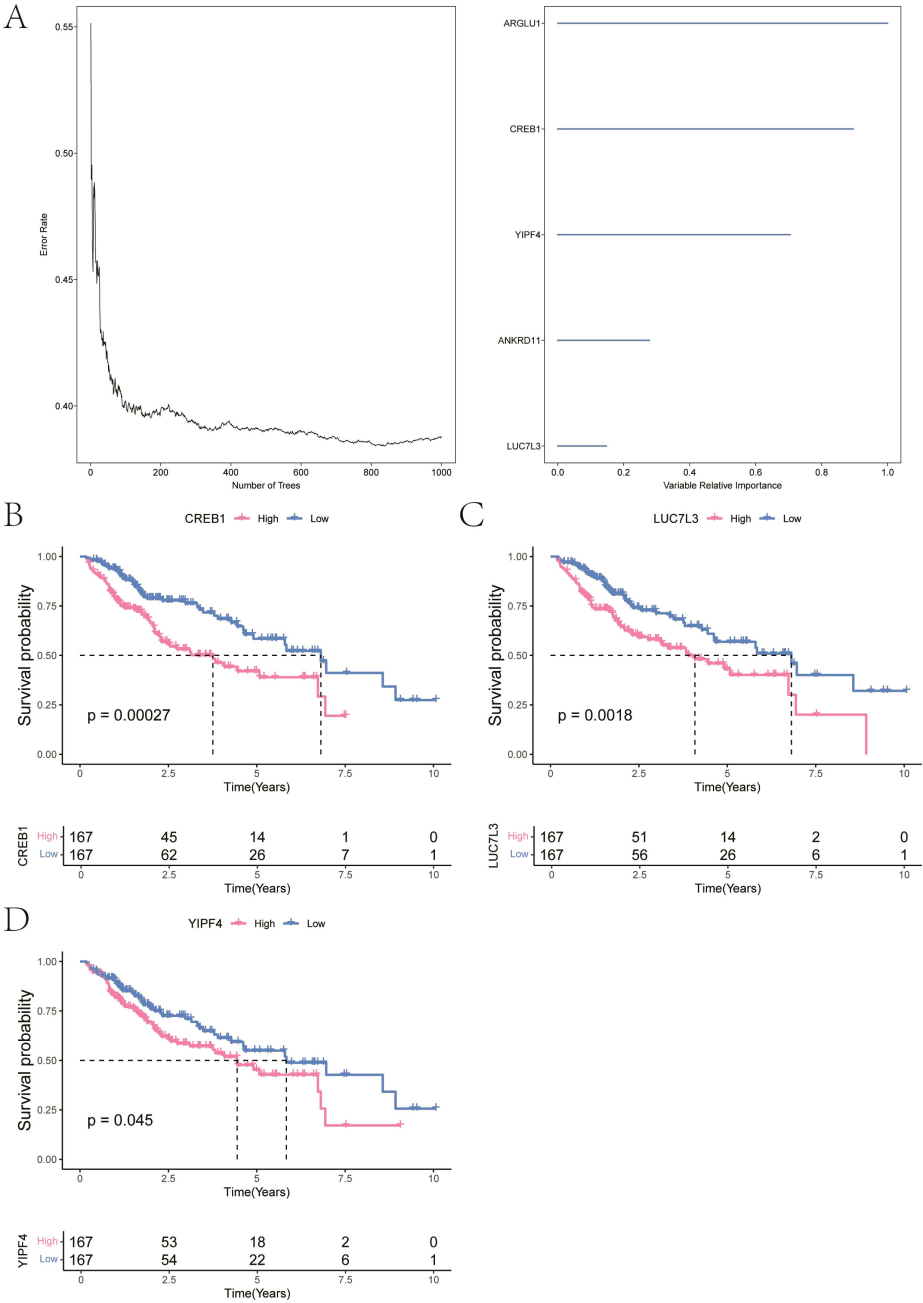
Identification of key fibrosis-related genes in HCC. **(A)** Random survival forest ranking of important genes. **(B–D)** Kaplan-Meier survival curves for key genes, including LUC7L3, CREB1, and YIPF4.

In order to further determine the fibrosis genes in HCC, we first obtained 38 marker genes from our single-cell RNA-seq differential expression analysis. These genes were intersected with differentially expressed genes from the TCGA-LIHC cohort, yielding 37 overlapping genes, which were then subjected to random survival forest analysis. With relative importance score greater than 0.1 as the significant markers, **Figure 5A** showed the order of importance of five identified genes. We then performed survival analysis of these five genes of high importance, and the survival curves showed that three genes were significantly associated with overall survival (p < 0.05) (**Figures 5B–D**). These genes included LUC7L3, CREB1, and YIPF4, which were identified as key genes for subsequent analysis.

### 3.4 Correlation analysis between key genes and fibrosis-related genes

**Figure 6:**
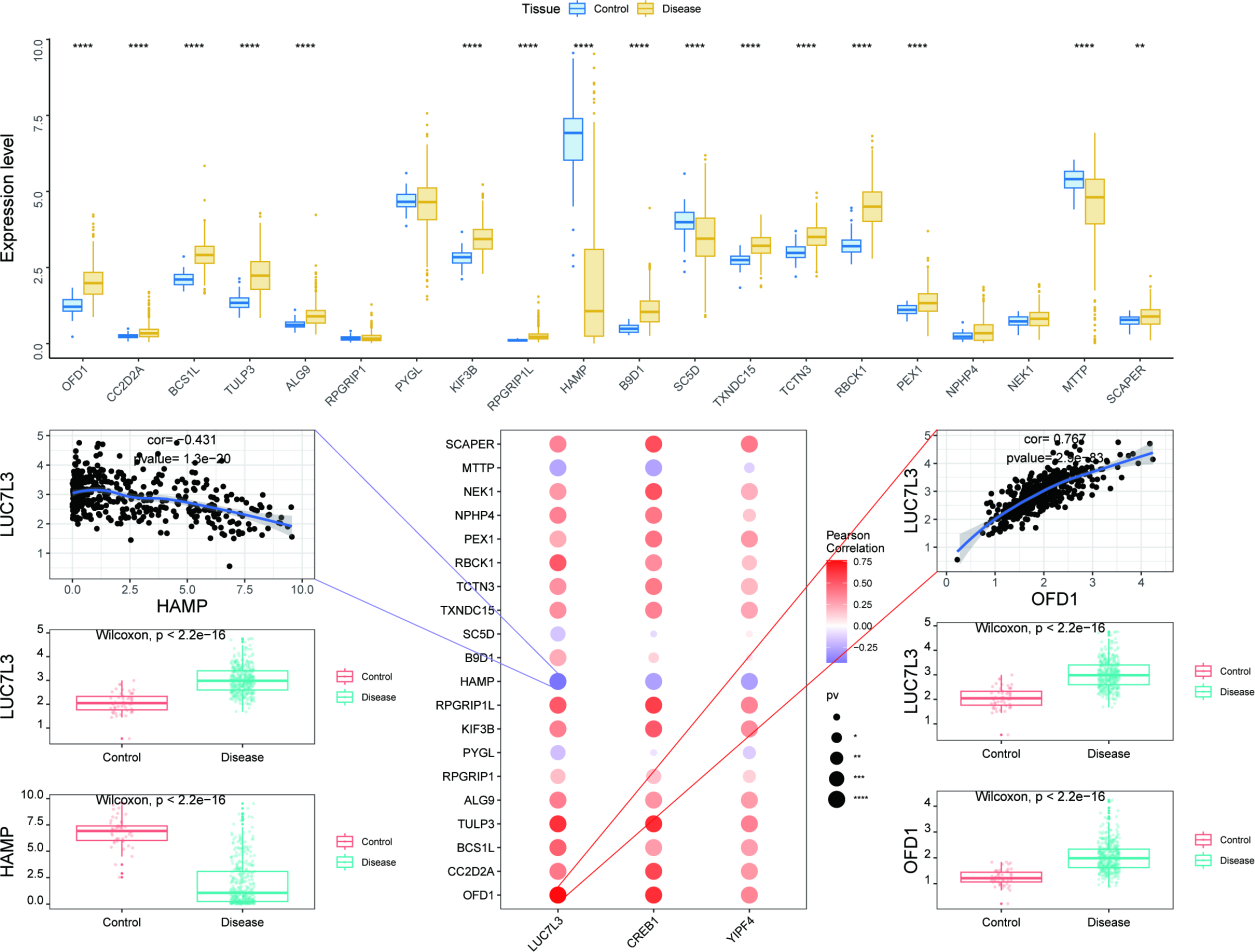
Expression and correlation analysis of fibrosis-related genes. **(A)** Expression levels of 20 fibrosis-related genes between control and disease groups. **(B)** Correlation analysis between fibrosis-related genes and three key genes. ** P < 0.01; **** P < 0.0001.

We obtained fibrosis-related genes from the MSigDB database, and the expression differences of fibrosis-related genes were analyzed (**Figure 6**). Twenty representative genes with the highest expression abundance and significant differential expression were selected for subsequent analysis to ensure biological relevance. Among them, sixteen genes showed significant differences between disease and control groups, including OFD1, CC2D2A, BCS1L, TULP3, ALG9, KIF3B, RPGRIP1L, HAMP, B9D1, SC5D, TXNDC15, TCTN3, RBCK1, PEX1, MTTP, and SCAPER. Subsequently, we performed correlation analysis between key genes and twenty fibrosis-related genes, and found that the expression levels of key genes were significantly correlated with the expression levels of some fibrosis-related genes. Notably, LUC7L3 and OFD1 were significantly positively correlated (r = 0.767), and it showed a negatively correlated with HAMP (r = −0.431).

Additionally, we also chose some fibrosis-related genes in MsigDB database, and investigated the co-expression situation across them and three key genes (**Supplementary Figures S2**-**S4**). All key genes were significantly positively correlated with OFD1 and KIF3B. As for the expression of TULP3, it was positively associated with LUC7L3, but negatively correlated with CREB1 and YIPF4. Then, “AUCell” algorithm was used to quantify the genes related to immunometabolism in single cells, and expression differences were visualized using bubble plots (**Supplementary Figure S5**). Specifically, CREB1 showed high activity in oxidative_phosphorylation, myc_targets_v1, and tnfa_signaling_via_nfkb. LUC7L3 was enriched in oxidative_phosphorylation, reactive_oxygen_species_pathway, and myc_targets_v1, while YIPF4 had high activity in oxidative_phosphorylation, reactive_oxygen_species_pathway, and coagulation.

### 3.5 Tumor immune microenvironment analysis

**Figure 7:**
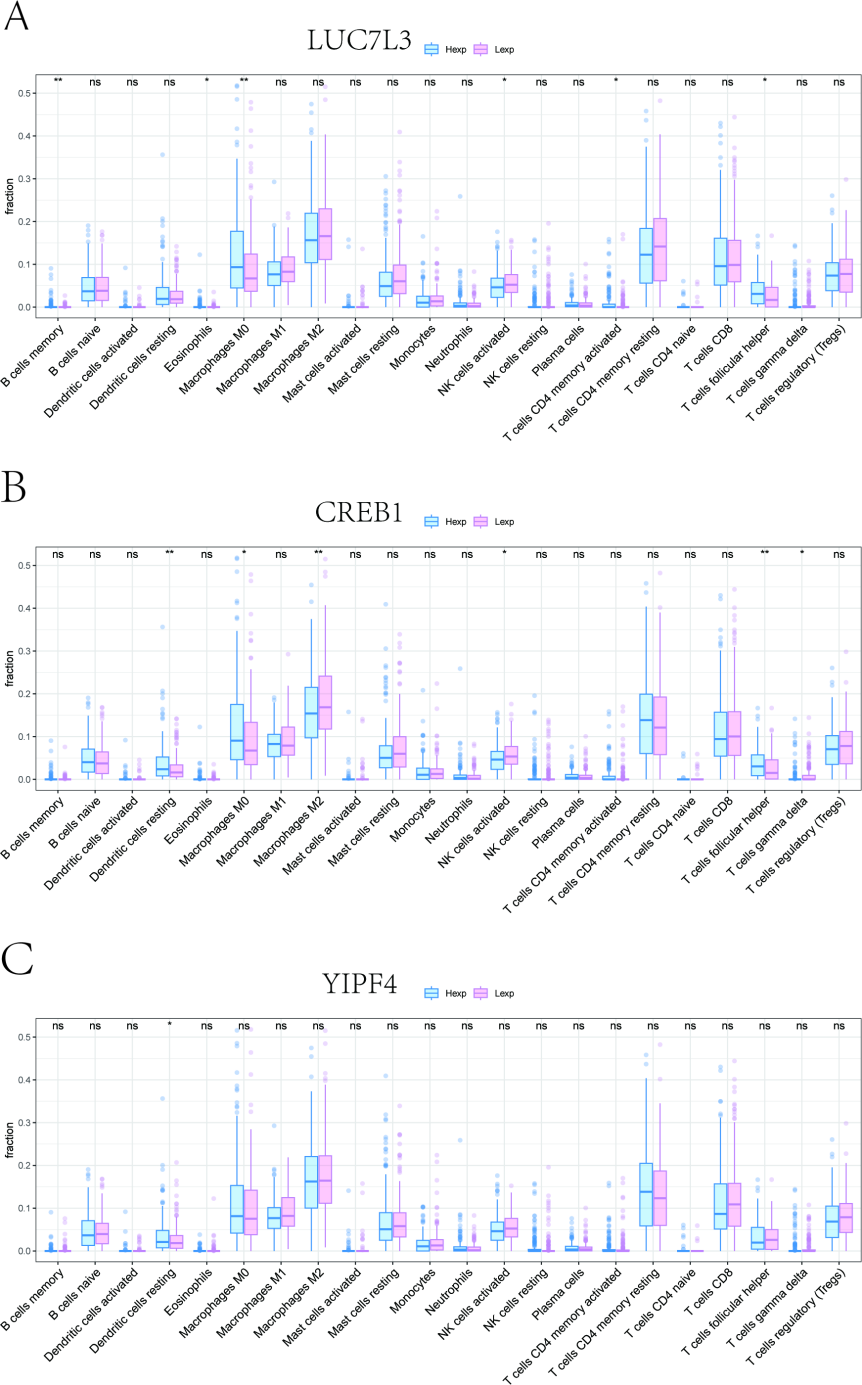
Tumor immune microenvironment analysis. **(A)** Immune cell fractions between high-expression group and low-expression group of LUC7L3. **(B)** Immune cell fractions between high-expression group and low-expression group of CREB1. **(C)** Immune cell fractions between high-expression group and low-expression group of YIPF4. * P < 0.05; ** P < 0.01; ns, not significant.

To explore how key genes may affect the progression of HCC, our study examined their correlations with immune-related cells. Specifically, we investigated the fractions of various immune cell populations between high-expression and low-expression groups of three key genes. It is note that the high-expression group of LUC7L3 was higher in macrophages M0 and T cells follicular helper cells, and the low-expression group was higher in NK cells activated and other cells (**Figure 7A**). As for CREB1, the high expression group was higher in dendritic cells resting, macrophages M0, and T cells follicular helper, while the low expression group was higher in macrophages M2, NK cells activated and other cells (**Figure 7B**). The high expression group of YIPF4 was higher in dendritic cells resting cells (**Figure 7C**).

### 3.6 GSEA and GSVA

**Figure 8:**
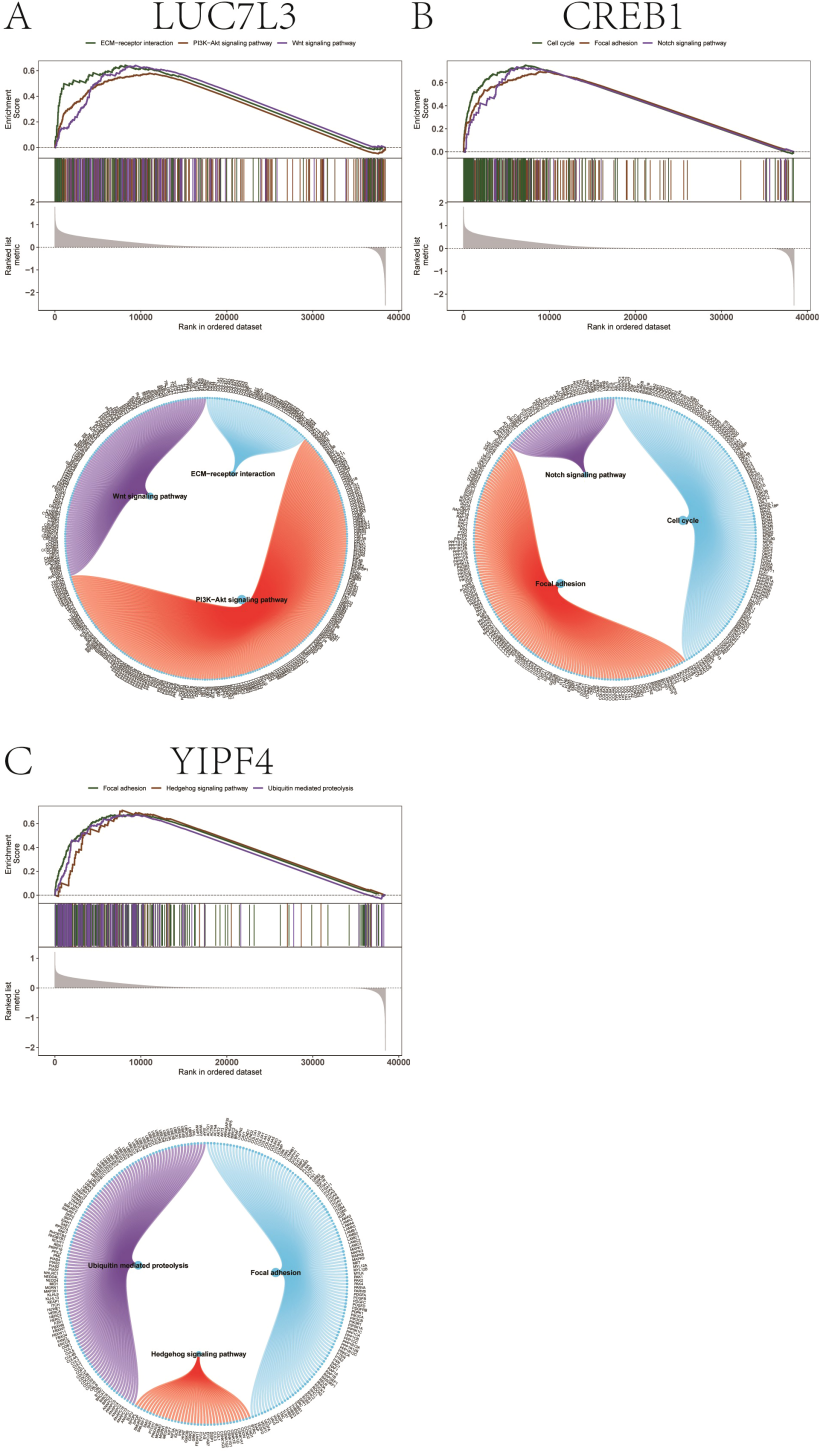
GSEA enrichment analysis of key genes. **(A)** Related pathways of LUC7L3. **(B)** Related pathways of CREB1. **(C)** Related pathways of YIPF4.

Furthermore, we analyzed the specific signaling pathways associated with key genes and explored the underlying molecular mechanisms through which key genes may influence the disease progression. The results of GSEA showed that LUC7L3 was enriched in Wnt signaling pathway, PI3K-Akt signaling pathway, and ECM-receptor interaction (**Figure 8A**). CREB1 was enriched in Cell cycle, Focal adhesion, and Notch signaling pathway, while YIPF4 was enriched in Focal adhesion, Hedgehog signaling pathway, and Ubiquitin mediated proteolysis (**Figures 8B, C**).

**Figure 9:**
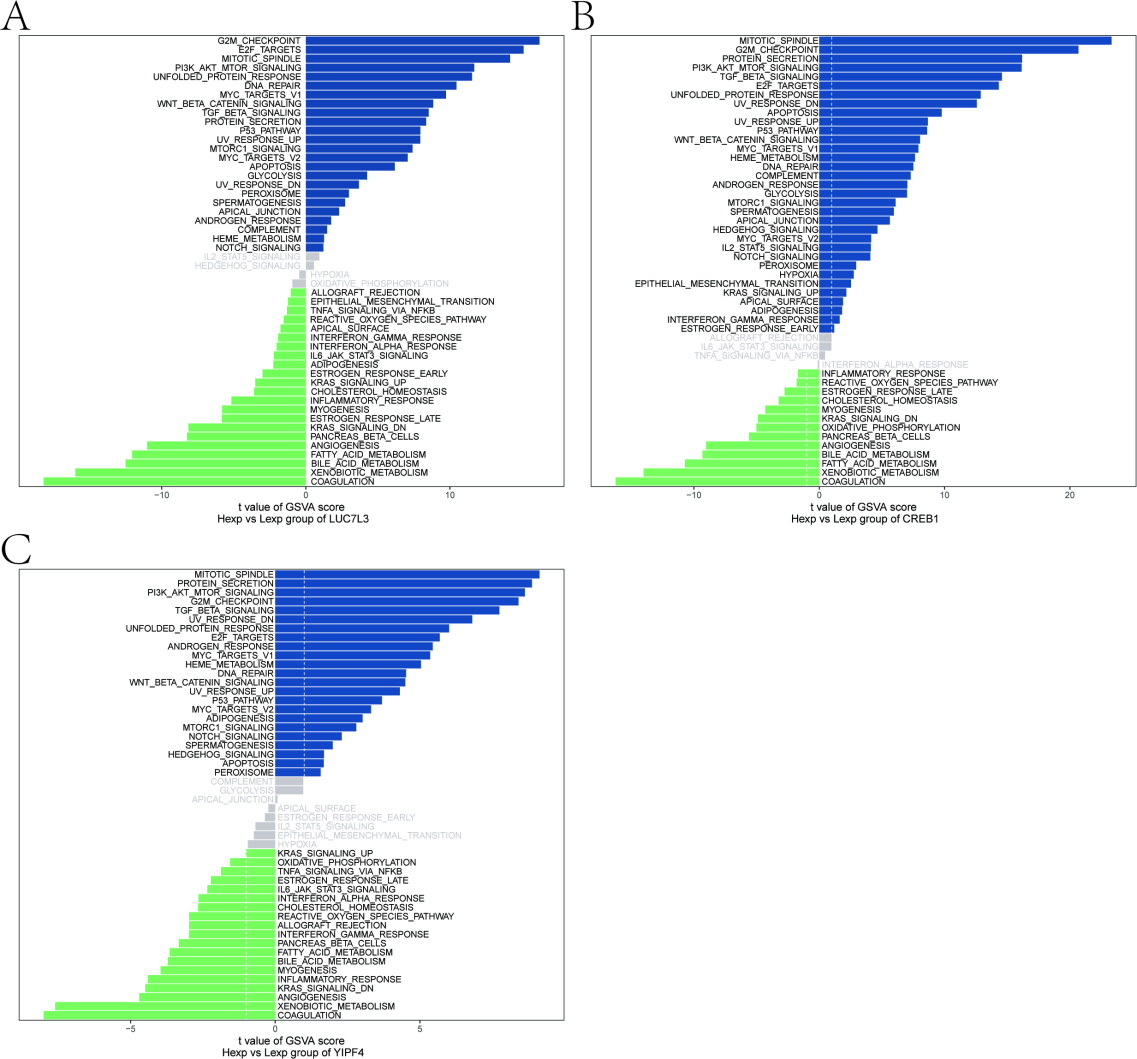
GSVA enrichment analysis of key genes. **(A)** Pathway differences between high-expression group and low-expression group of LUC7L3. **(B)** Pathway differences between high-expression group and low-expression group of CREB1. **(C)** Pathway differences between high-expression group and low-expression group of YIPF4.

The results of GSVA indicated that the COAGULATION pathway was predominantly active in the low-expression groups of three key genes (**Figure 9**). In the high-expression groups, enrichment was observed in pathways including the G2M_CHECKPOINT, MITOTIC_SPINDLE, and others, implying that key genes may contribute to disease progression via these different pathways.

### 3.7 Drug susceptibility analysis

**Figure 10:**
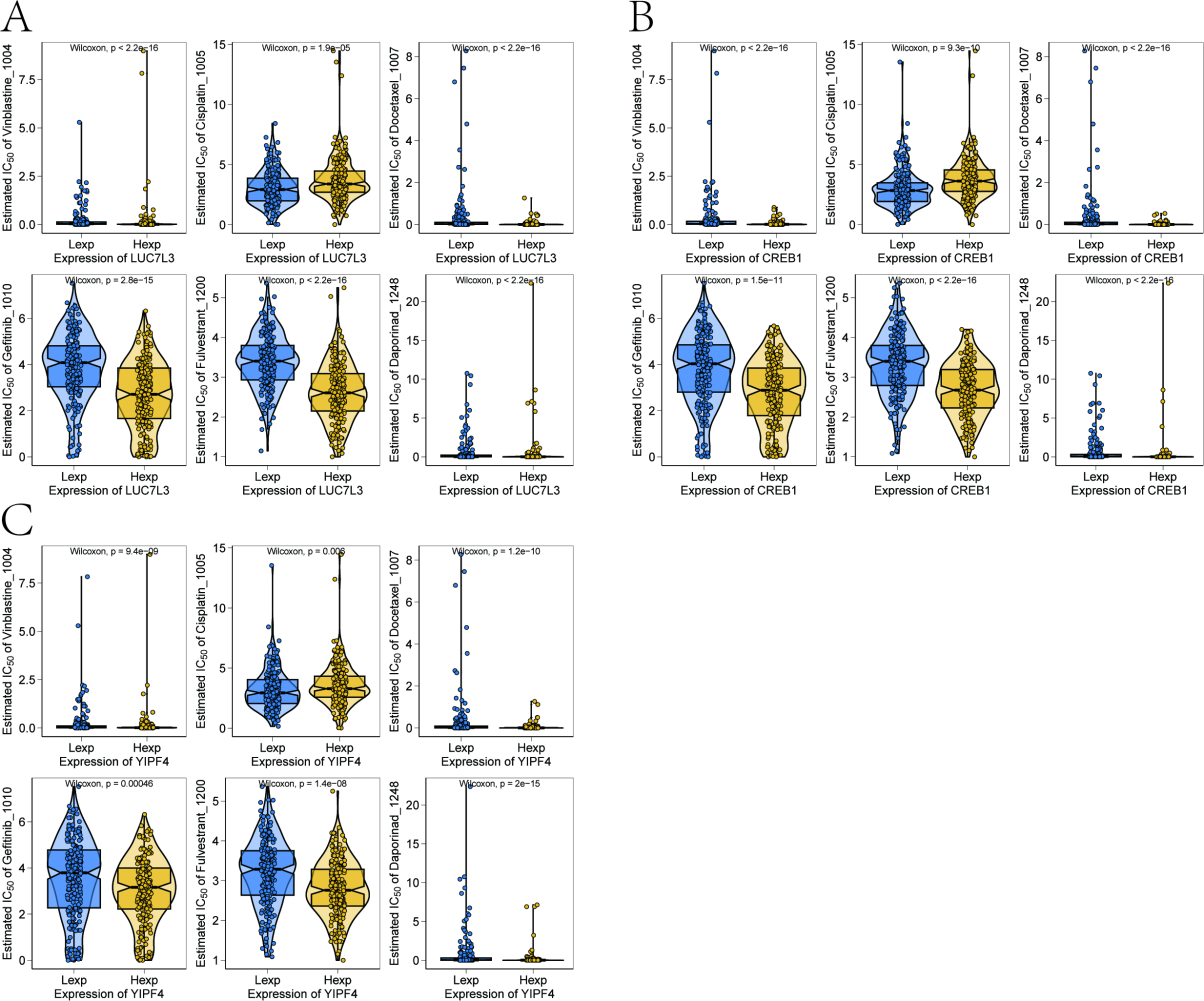
Drug susceptibility analysis of key genes. **(A)** Chemotherapy response prediction for LUC7L3. **(B)** Chemotherapy response prediction for CREB1. **(C)** Chemotherapy response prediction for YIPF4.

According to the drug sensitivity data from the GDSC database, our study employed the “oncoPredict” package to predict chemotherapy sensitivity in each tumor sample, and investigated the responses of the three key genes and to common chemotherapeutic drugs (**Figure 10**). The sensitivity assessment of chemotherapeutic drugs showed that key genes were correlated with Vinblastine_1004, Cisplatin_1005, Docetaxel_1007, Gefitinib_1010, Fulvestrant_1200 and Daporinad_1248.

### 3.8 Spatial transcriptomics analysis

**Figure 11:**
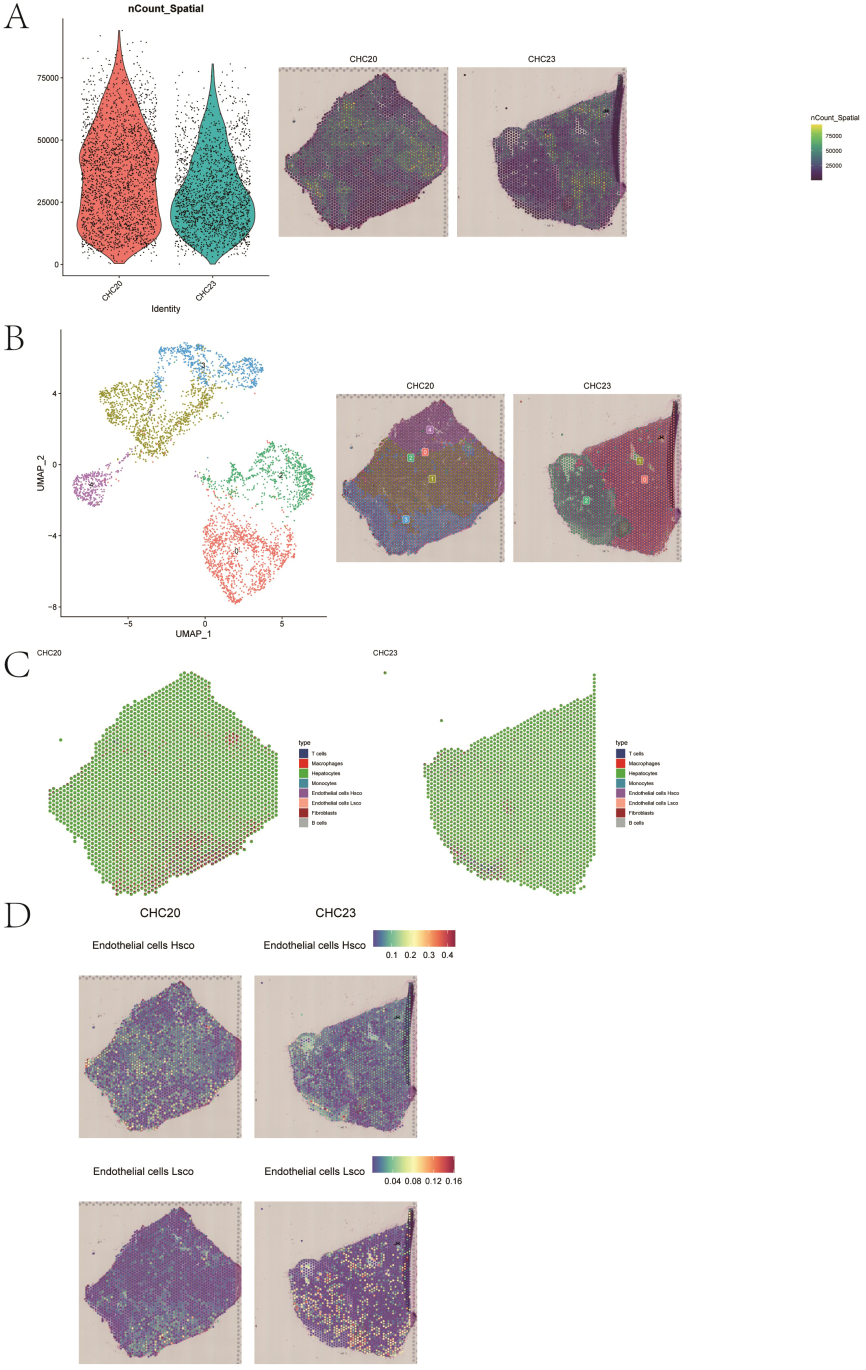
Spatial transcriptomic analysis and cell composition mapping. **(A, B)** UMAP visualization and clustering of two spatial transcriptomic samples. **(C, D)** Spatial distribution of cell populations stratified by fibrosis score.

We sequentially read two spatial transcriptomic samples and examined the distribution of UMI counts. After data normalization, PCA and UMAP were applied for dimensionality reduction. Subsequent Louvain clustering identified five distinct cell subpopulations across the two spatial transcriptomic samples (**Figures 11A, B**). We then performed deconvolution analysis of the spatial transcriptomic data in combination with single-cell data to estimate the cellular composition of each spot. Additionally, we visualized the spatial distribution of key cell populations stratified by high and low fibrosis scores within the spatial transcriptome (**Figures 11C, D**).

**Figure 12:**
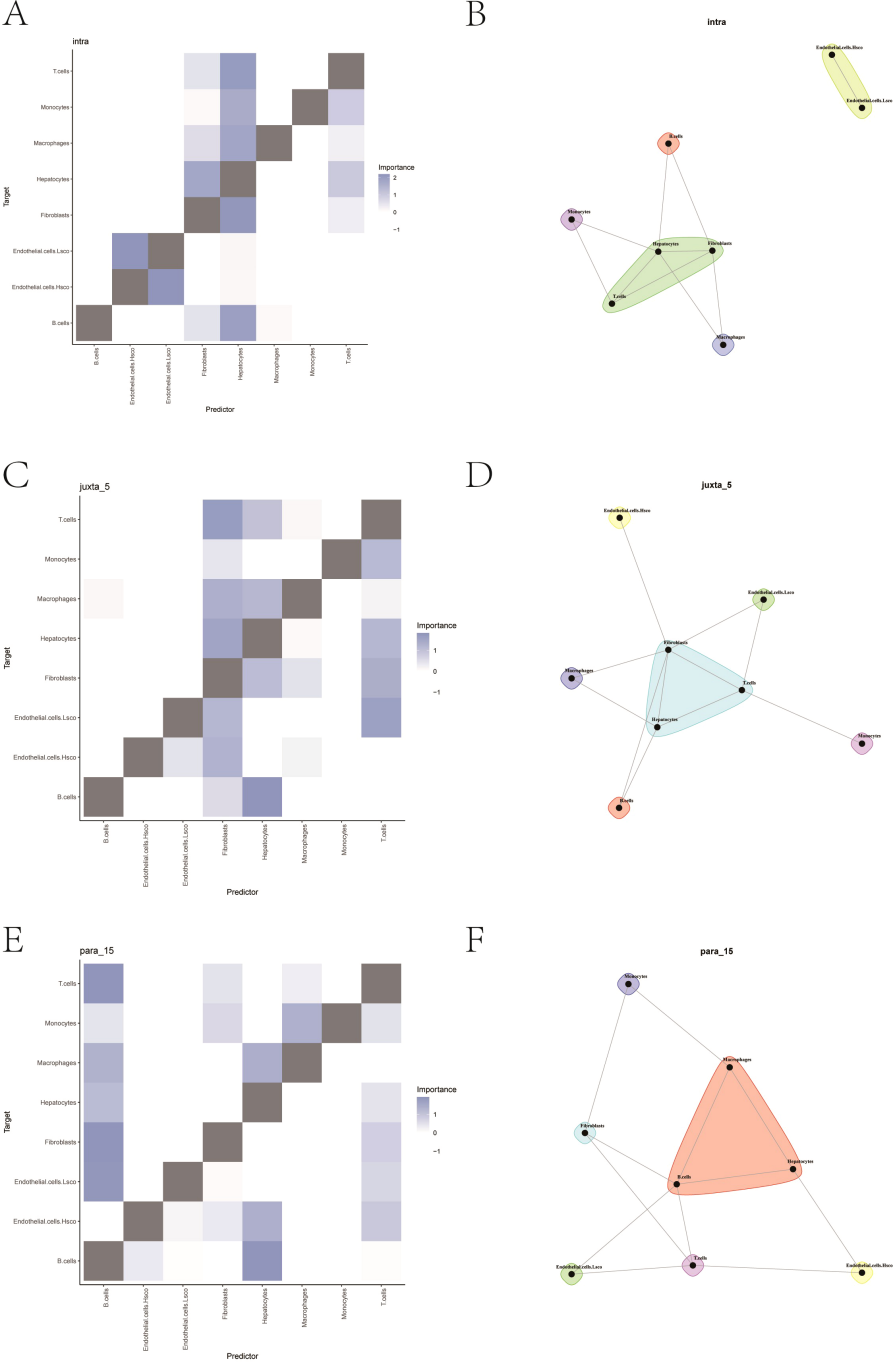
Spatial modeling of intercellular communication using MISTy. **(A, C, E)** Heatmaps showing interaction strength across annotated cell types. **(B, D, F)** Predicted interaction networks between each cell subgroups for each sample.

**Figure 13:**
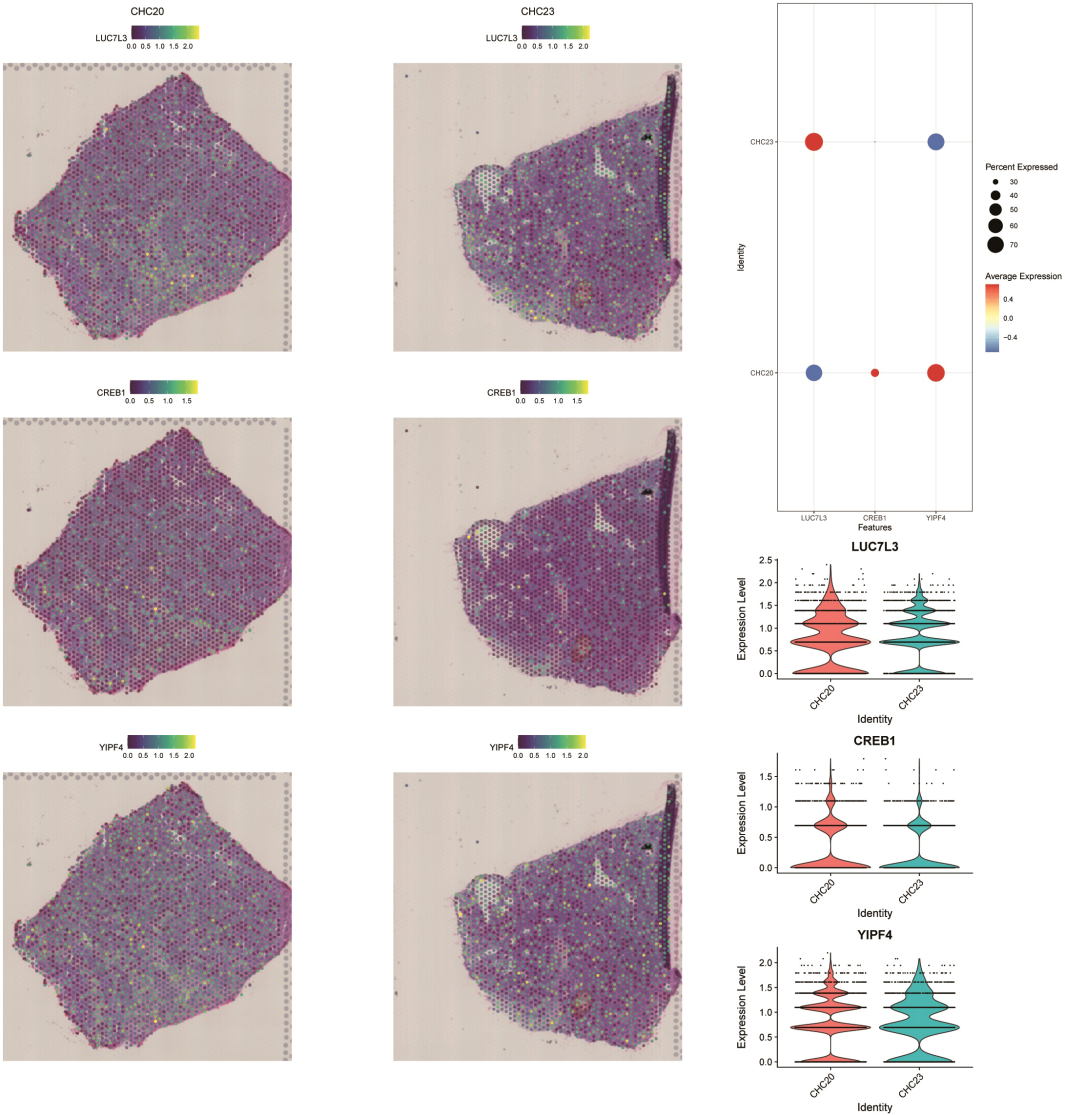
Spatial expression of key genes in liver tissue, including LUC7L3, CREB1, and YIPF4.

In order to gain a deeper understanding of intracellular and intercellular interactions, we employed the MISTy. By analyzing interactions based on the annotated cell identities, we generated heatmaps and network diagrams of intercellular communication (**Figure 12**). The spatial expression patterns of the key genes were further analyzed, and the expression levels of LUC7L3, CREB1, and YIPF4 were visualized across the two spatial transcriptomic datasets (**Figure 13**).

**Figure 14:**
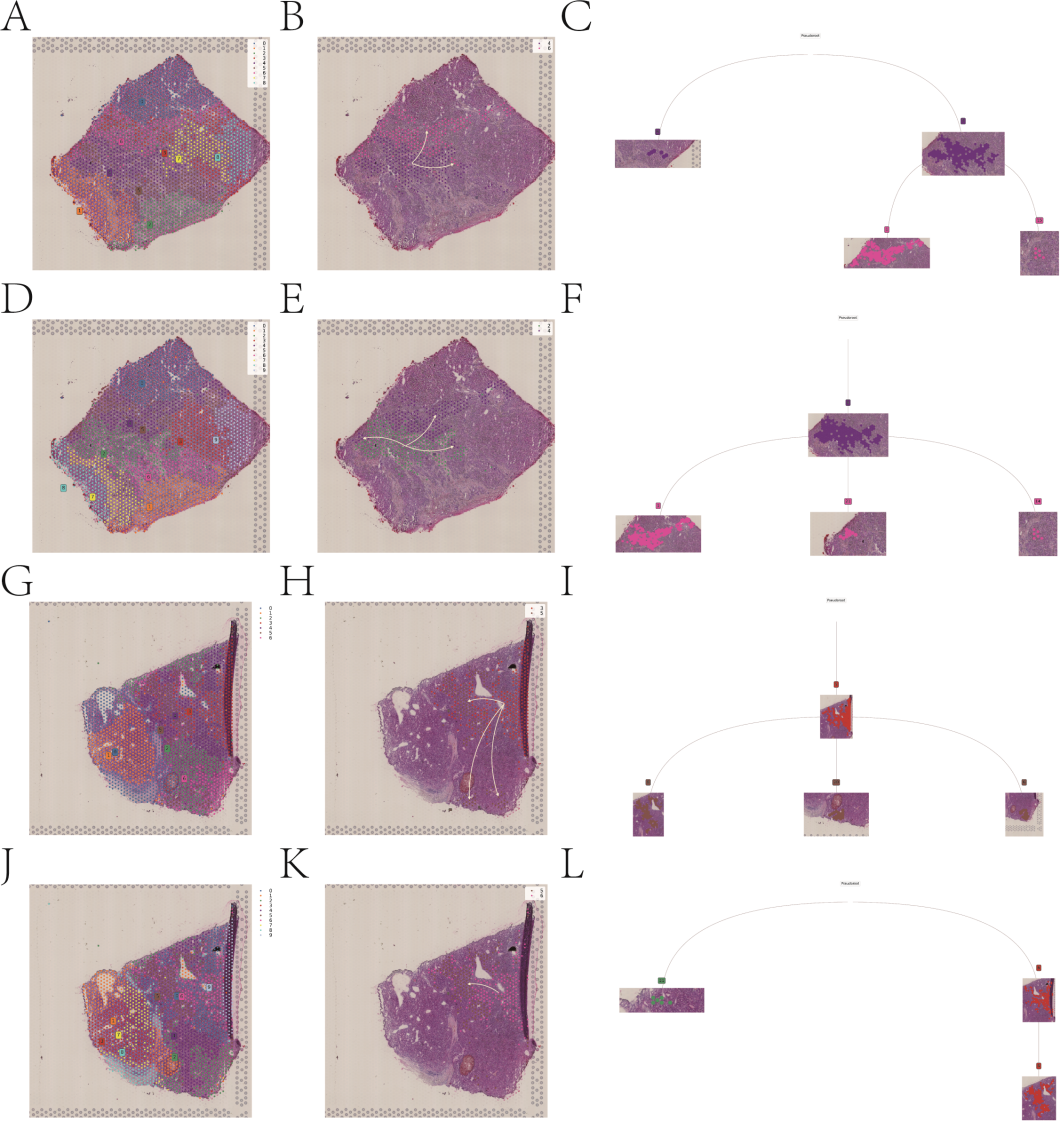
Spatial trajectory analysis of cellular dynamics using STlearn.

Finally, STlearn was applied to investigate the spatial distribution of cell types and their interactions in HCC tissues. The spatial trajectory analysis revealed a clear directional cellular transition beginning at the tumor boundary and progressing toward the fibrosis- and necrosis-enriched tumor core regions (**Figure 14**). This spatial transition pattern suggests coordinated remodeling among major cell clusters during HCC progression.

### 3.9 Validation of the key gene

**Figure 15:**
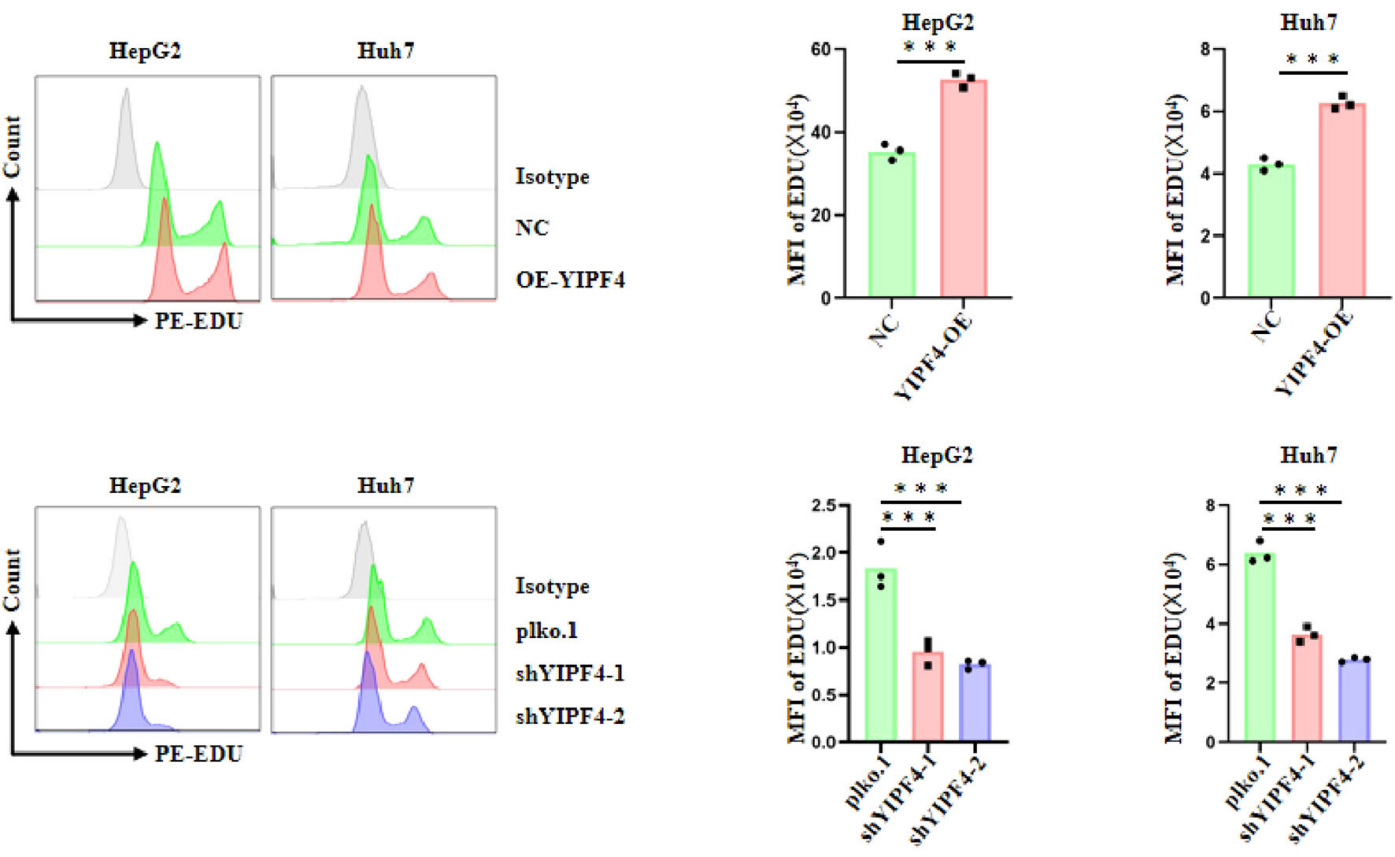
EDU incorporation assay for YIPF4 in HCC cell lines.

Previous studies have experimentally shown that LUC7L3 and CREB1 are involved in the progression of HCC (37, 38); therefore, we further focused on YIPF4 for experimental validation. To investigate the role of YIPF4 in HCC cell proliferation and migration, we performed EDU incorporation assay, Transwell migration assay, and CCK-8 proliferation assay in HepG2 and Huh7 cell lines. Over-expression of YIPF4 significantly increased EDU fluorescence intensity, indicating enhanced DNA synthesis and cell proliferation, whereas knockdown of YIPF4 markedly reduced EDU incorporation (**Figure 15**). Moreover, Transwell assay demonstrated that up-regulation of YIPF4 led to a significant increase in the number of migrated cells, whereas silencing YIPF4 suppressed cell migration (**Supplementary Figure S6**). Consistently, the results of CCK-8 assay showed that YIPF4 over-expression promoted cell viability in a time-dependent manner, while YIPF4 knockdown impaired proliferative capacity (**Supplementary Figure S7**). In conclusion, these results suggest that YIPF4 promotes both proliferation and migration of HCC cells.

## 4 Discussion

HCC exhibits a high degree of heterogeneity, characterized by diverse morphological features and biological behaviors (39). Clinically, despite the advancement of early diagnostic technologies, it is often diagnosed at a late stage due to its asymptomatic onset (40). Current treatment options, including surgical resection, local ablation, and liver transplantation remain limited for patients with advanced stages (41). Although systemic therapies, such as targeted therapy and immunotherapy, have shown clinical benefits in some patients, the variability in immune response and the complex TME present major challenges, necessitating further exploration of novel therapeutic targets and strategies (42–45). Liver fibrosis, an end-stage liver disease, is characterized by excessive accumulation of fibrous tissue (46). It not only disrupts normal liver physiology but also significantly increases the risk of HCC (47). However, there is still a lack of relevant research on fibrosis-related genes and their roles in HCC progression. Thus, key genes were identified through GEO and TCGA datasets, with YIPF4 further validated by cellular experiments.

In recent years, immunotherapy for HCC has made significant progress, particularly the application of immune checkpoint inhibitors, which have shown promising efficacy in certain patients (48). HCC is characterized by a highly immunosuppressive microenvironment in which tumor cells evade immune surveillance through various mechanisms (49–53). For instance, HCC cells may express PD-L1, binding to the PD-1 receptor on T cells and suppressing their activity, thereby escaping immune clearance (54). In addition, immunosuppressive infiltrating cells, such as regulatory T cells and tumor-associated macrophages, facilitate tumor progression and metastasis (55, 56). Therefore, enhancing immunity in HCC requires not only blocking immune checkpoint pathways, but also addressing the immunosuppressive components of the TME. Our findings helped form the foundation for this, and contributed novel insights into HCC diagnosis, therapies, and prognosis.

Recent bioinformatics and molecular studies have provided valuable insights into the genetic and signaling mechanisms underlying HCC progression (13, 51, 57–63), paving the way for single-cell and spatial transcriptomic analyses to further dissect tumor heterogeneity. The advent of single-cell RNA sequencing has significantly advanced the understanding of the cellular composition and gene expression dynamics within the TME (64). It reveals the heterogeneity and distinct cellular sub-populations present in tumors, enabling precise characterization and quantification of immune cell infiltration in tumor tissues (52, 65, 66). Spatial transcriptomics, as an important advancement in single-cell sequencing, allows precise localization of gene expression within tissue sections (67, 68). This technology enables the direct observation of spatial gene expression patterns, revealing the heterogeneity of tumor tissues, the immune microenvironment, and intercellular interactions (69). We combined single-cell RNA sequencing with spatial transcriptomics to validate the expression patterns of key genes in liver cancer tissues and their relationships with the TME. It demonstrated that these genes exhibit distinct spatial expression patterns and are closely associated with HCC development and immune evasion. This integrated approach enhanced our understanding of the molecular mechanisms of HCC and provided a reliable basis for early diagnosis and targeted therapy. Spatial transcriptomics further complemented the single-cell analysis by validating the spatial distribution of fibrosis-related cell clusters and key genes within HCC tissues. Notably, LUC7L3, CREB1, and YIPF4 were mainly localized in endothelial- and fibrosis-rich regions, particularly at the tumor–stroma boundary, consistent with the single-cell findings. Moreover, the integration of MISTy and STlearn analyses provided additional evidence of cell–cell communication and spatial trajectories that underlie fibrosis-associated tumor progression. In addition, our single-cell interaction analysis revealed that endothelial cells with high fibrosis activity showed enhanced communication with macrophages via the MIF–CD74 signaling axis. This crosstalk may promote macrophage activation and TGF-β secretion, leading to endothelial activation, angiogenesis, and extracellular matrix deposition. These findings suggest that endothelial–macrophage interactions through MIF–CD74 contribute to fibrotic remodeling in the HCC microenvironment.

In this study, we identified fibrosis-related genes associated with HCC, with a focus on LUC7L3, CREB1, and YIPF4. Although the role of LUC7L3 in HCC remains poorly understood, existing evidence implicates it in the pathogenesis of various cancers (37). LUC7L3 functions as a regulator of RNA splicing and has been associated with cell proliferation and migration (70). Bioinformatic analysis and published evidence suggest that LUC7L3 may promote HCC cell proliferation and metastatic potential, indicating its possible contribution to tumor progression through regulatory effects on proliferation, migration, and invasion (37). CREB1 is a transcription factor broadly involved in the regulation of cell proliferation, differentiation, and survival. It is significantly up-regulated in HCC and is associated with poor prognosis and tumor progression (71). CREB1 promotes HCC cell growth and metastasis by activating key signaling pathways, including PI3K and MAPK, thereby underscoring its critical role in tumor progression and immune evasion (72). YIPF4, a gene implicated in membrane trafficking, has an unclear role in HCC (73, 74). In our study, we found that YIPF4 over-expression significantly promoted HCC cell proliferation and metastasis, as demonstrated by CCK-8, Transwell, and EdU assays. These findings suggest that YIPF4 may contribute to HCC progression by modulating key cellular functions. Therefore, YIPF4 could represent a therapeutic target for HCC, particularly in microtubule-targeting treatments, and holds promise for advancing precision oncology strategies. Notably, LUC7L3 showed a strong positive correlation with OFD1, suggesting that aberrant RNA splicing may be linked to centriole- and cilia-related processes involved in extracellular matrix remodeling. Conversely, its negative correlation with HAMP implies potential crosstalk between iron metabolism and fibrosis-associated transcriptional programs. These relationships provide mechanistic clues for how fibrosis-related genes may coordinate tumor progression and immune modulation in HCC. Although the present study identified key fibrosis-related genes with important mechanistic and prognostic implications in HCC, it is important to acknowledge potential heterogeneity across different disease etiologies. Viral-related HCC (e.g., HBV/HCV) and non-viral HCC (e.g., NASH, alcohol-related) display distinct immune microenvironment characteristics and fibrosis patterns, which may influence the expression and functional roles of genes such as LUC7L3, CREB1, and YIPF4. Due to dataset limitations, subgroup analyses stratified by etiology were not feasible in this study. Future studies incorporating larger cohorts with well-defined etiological backgrounds are needed to validate the applicability of these findings across diverse patient populations.

Although our study identified and validated fibrosis-related genes using single-cell and spatial transcriptomics, certain limitations remain. Firstly, while the functional roles of key genes were confirmed in cells, further validation using in animal models is necessary to assess their therapeutic potential. Secondly, although these genes are implicated in HCC, their specific molecular mechanisms within the immune microenvironment of HCC require further investigation. Future studies should focus on the interaction between these genes and immune cells, as well as their roles in immunotherapy. Additionally, challenges also remain in data interpretation and analysis. Further optimization of analytical methods is needed to enhance data reliability and accuracy. In addition, while our functional experiments were performed using HepG2 and Huh7, two widely used HCC cell models with distinct biological characteristics, future studies incorporating additional cell lines may help further validate the generalizability of these findings. Lastly, the sample size in this study was limited, and further validation through prospective, large-scale studies is warranted to confirm these findings. Future studies will further validate these in silico drug sensitivity predictions through cell-based assays and pharmacological experiments. This study identified key genes related to liver fibrosis and HCC through integrated single-cell and spatial transcriptomic analyses, and revealed their potential roles in tumor progression and immune evasion. These findings had important implications and may serve as a basis for the development of novel therapeutic targets, particularly in the context of immunotherapy.

## 5 Conclusion

By integrating single-cell and spatial transcriptomic analyses, we identified fibrosis-related prognostic genes in HCC, among which YIPF4 played a key role in promoting tumor cell proliferation and migration. We also elaborated on the role of these prognostic genes in the tumor immune microenvironment. These findings shed light on the molecular link between fibrosis and HCC progression, offering potential targets for therapeutic intervention.

## Data Availability

The original contributions presented in the study are included in the article/**Supplementary Material**. Further inquiries can be directed to the corresponding authors.

## Supplementary material

Supplementary Figure 1 The quality control before and after filtration.

Supplementary Figure 2 Co-expression analysis between fibrosis-related genes and LUC7L3. **(A)** Co-expression analysis between OFD1 and LUC7L3. **(B)** Co-expression analysis between TULP3 and LUC7L3. **(C)** Co-expression analysis between KIF3B and LUC7L3.

Supplementary Figure 3 Co-expression analysis between fibrosis-related genes and CREB1. **(A)** Co-expression analysis between OFD1 and CREB1. **(B)** Co-expression analysis between TULP3 and CREB1. **(C)** Co-expression analysis between KIF3B and CREB1.

Supplementary Figure 4 Co-expression analysis between fibrosis-related genes and YIPF4. **(A)** Co-expression analysis between OFD1 and YIPF4. **(B)** Co-expression analysis between TULP3 and YIPF4. **(C)** Co-expression analysis between KIF3B and YIPF4.

Supplementary Figure 5 Immunometabolism-related pathways for three key genes.

Supplementary Figure 6 Transwell migration assay for YIPF4 in HCC cell lines.

Supplementary Figure 7 CCK-8 proliferation assay for YIPF4 in HCC cell lines.

## References

[1] Bray F Ferlay J Soerjomataram I Siegel RL Torre LA Jemal A . Global cancer statistics 2018: GLOBOCAN estimates of incidence and mortality worldwide for 36 cancers in 185 countries. CA: Cancer J Clin. (2018) 68:394–424. doi: 10.3322/caac.21492, PMID: 30207593

[2] Torre LA Siegel RL Ward EM Jemal A . Global cancer incidence and mortality rates and trends–an update. Cancer epidemiology Biomarkers Prev. (2016) 25:16–27. doi: 10.1158/1055-9965.Epi-15-0578, PMID: 26667886

[3] Altaf S Saleem F Sher AA Ali A . Potential therapeutic strategies to combat HCC. Curr Mol Pharmacol. (2022) 15:929–42. doi: 10.2174/1874467215666220103111009, PMID: 34979895

[4] Du Y Liu D Du Y . Recent advances in hepatocellular carcinoma therapeutic strategies and imaging-guided treatment. J Drug Targeting. (2022) 30:287–301. doi: 10.1080/1061186x.2021.1999963, PMID: 34727794

[5] Sangro B Sarobe P Hervás-Stubbs S Melero I . Advances in immunotherapy for hepatocellular carcinoma. Nat Rev Gastroenterol Hepatol. (2021) 18:525–43. doi: 10.1038/s41575-021-00438-0, PMID: 33850328 PMC8042636

[6] Oura K Morishita A Tani J Masaki T . Tumor immune microenvironment and immunosuppressive therapy in hepatocellular carcinoma: A review. Int J Mol Sci. (2021) 22. doi: 10.3390/ijms22115801, PMID: 34071550 PMC8198390

[7] Parola M Pinzani M . Liver fibrosis: Pathophysiology, pathogenetic targets and clinical issues. Mol aspects Med. (2019) 65:37–55. doi: 10.1016/j.mam.2018.09.002, PMID: 30213667

[8] Bataller R Brenner DA . Liver fibrosis. J Clin Invest. (2005) 115:209–18. doi: 10.1172/jci24282, PMID: 15690074 PMC546435

[9] Sotoudeheian M . Galectin-3 and severity of liver fibrosis in metabolic dysfunction-associated fatty liver disease. Protein Pept Lett. (2024) 31:290–304. doi: 10.2174/0109298665301698240404061300, PMID: 38715329

[10] Abril-Rodriguez G Ribas A . SnapShot: immune checkpoint inhibitors. Cancer Cell. (2017) 31:848–848.e1. doi: 10.1016/j.ccell.2017.05.010, PMID: 28609660

[11] De Martin E Fulgenzi CAM Celsa C Laurent-Bellue A Torkpour A Lombardi P . Immune checkpoint inhibitors and the liver: balancing therapeutic benefit and adverse events. Gut. (2024) 74:1165–77. doi: 10.1136/gutjnl-2024-332125, PMID: 39658265

[12] Gudivada IP Amajala KC . Integrative bioinformatics analysis for targeting hub genes in hepatocellular carcinoma treatment. Curr Genomics. (2025) 26:48–80. doi: 10.2174/0113892029308243240709073945, PMID: 39911278 PMC11793067

[13] Xu W Liao S Hu Y Huang Y Zhou J . Upregulation of miR-3130-5p Enhances Hepatocellular Carcinoma Growth by Suppressing Ferredoxin 1: miR-3130-5p Enhances HCC Growth via Inhibiting FDX1. Curr Mol Pharmacol. (2024) 17:e18761429358008. doi: 10.2174/0118761429358008250305070518, PMID: 40103455

[14] Harkus U Wankell M Palamuthusingam P McFarlane C Hebbard L . Immune checkpoint inhibitors in HCC: Cellular, molecular and systemic data. Semin Cancer Biol. (2022) 86:799–815. doi: 10.1016/j.semcancer.2022.01.005, PMID: 35065242

[15] Cheng AL Hsu C Chan SL Choo SP Kudo M . Challenges of combination therapy with immune checkpoint inhibitors for hepatocellular carcinoma. J Hepatol. (2020) 72:307–19. doi: 10.1016/j.jhep.2019.09.025, PMID: 31954494

[16] Li Z Chen B Dong W Kong M Fan Z Yu L . MKL1 promotes endothelial-to-mesenchymal transition and liver fibrosis by activating TWIST1 transcription. Cell Death Dis. (2019) 10:899. doi: 10.1038/s41419-019-2101-4, PMID: 31776330 PMC6881349

[17] Ruan B Duan JL Xu H Tao KS Han H Dou GR . Capillarized liver sinusoidal endothelial cells undergo partial endothelial-mesenchymal transition to actively deposit sinusoidal ECM in liver fibrosis. Front Cell Dev Biol. (2021) 9:671081. doi: 10.3389/fcell.2021.671081, PMID: 34277612 PMC8285099

[18] Qu J Wang L Li Y Li X . Liver sinusoidal endothelial cell: An important yet often overlooked player in the liver fibrosis. Clin Mol Hepatol. (2024) 30:303–25. doi: 10.3350/cmh.2024.0022, PMID: 38414375 PMC11261236

[19] Cheng C Chen W Jin H . Chen X. A review of single-cell RNA-seq annotation, integration, and cell-cell communication. Cells. (2023) 12. doi: 10.3390/cells12151970, PMID: 37566049 PMC10417635

[20] van Galen P Hovestadt V Wadsworth Ii MH Hughes TK Griffin GK Battaglia S . Single-cell RNA-seq reveals AML hierarchies relevant to disease progression and immunity. Cell. (2019) 176:1265–1281.e24. doi: 10.1016/j.cell.2019.01.031, PMID: 30827681 PMC6515904

[21] Rao A Barkley D França GS Yanai I . Exploring tissue architecture using spatial transcriptomics. Nature. (2021) 596:211–20. doi: 10.1038/s41586-021-03634-9, PMID: 34381231 PMC8475179

[22] Wang Y Liu B Zhao G Lee Y Buzdin A Mu X . Spatial transcriptomics: Technologies, applications and experimental considerations. Genomics. (2023) 115:110671. doi: 10.1016/j.ygeno.2023.110671, PMID: 37353093 PMC10571167

[23] Tian L Chen F Macosko EZ . The expanding vistas of spatial transcriptomics. Nat Biotechnol. (2023) 41:773–82. doi: 10.1038/s41587-022-01448-2, PMID: 36192637 PMC10091579

[24] Lu Y Yang A Quan C Pan Y Zhang H Li Y . A single-cell atlas of the multicellular ecosystem of primary and metastatic hepatocellular carcinoma. Nat Commun. (2022) 13:4594. doi: 10.1038/s41467-022-32283-3, PMID: 35933472 PMC9357016

[25] Giraud J Chalopin D Ramel E Boyer T Zouine A Derieppe MA . THBS1(+) myeloid cells expand in SLD hepatocellular carcinoma and contribute to immunosuppression and unfavorable prognosis through TREM1. Cell Rep. (2024) 43:113773. doi: 10.1016/j.celrep.2024.113773, PMID: 38350444

[26] Stuart T Butler A Hoffman P Hafemeister C Papalexi E Mauck WM . Comprehensive integration of single-cell data. Cell. (2019) 177:1888–1902.e21. doi: 10.1016/j.cell.2019.05.031, PMID: 31178118 PMC6687398

[27] McGinnis CS Murrow LM Gartner ZJ . DoubletFinder: doublet detection in single-cell RNA sequencing data using artificial nearest neighbors. Cell Syst. (2019) 8:329–337.e4. doi: 10.1016/j.cels.2019.03.003, PMID: 30954475 PMC6853612

[28] Korsunsky I Millard N Fan J Slowikowski K Zhang F Wei K . Fast, sensitive and accurate integration of single-cell data with Harmony. Nat Methods. (2019) 16:1289–96. doi: 10.1038/s41592-019-0619-0, PMID: 31740819 PMC6884693

[29] Jin S Guerrero-Juarez CF Zhang L Chang I Ramos R Kuan CH . Inference and analysis of cell-cell communication using CellChat. Nat Commun. (2021) 12:1088. doi: 10.1038/s41467-021-21246-9, PMID: 33597522 PMC7889871

[30] Taylor JM . Random survival forests. J Thorac Oncol. (2011) 6:1974–5. doi: 10.1097/JTO.0b013e318233d835, PMID: 22088987

[31] Newman AM Liu CL Green MR Gentles AJ Feng W Xu Y . Robust enumeration of cell subsets from tissue expression profiles. Nat Methods. (2015) 12:453–7. doi: 10.1038/nmeth.3337, PMID: 25822800 PMC4739640

[32] Subramanian A Tamayo P Mootha VK Mukherjee S Ebert BL Gillette MA . Gene set enrichment analysis: a knowledge-based approach for interpreting genome-wide expression profiles. Proc Natl Acad Sci U S A. (2005) 102:15545–50. doi: 10.1073/pnas.0506580102, PMID: 16199517 PMC1239896

[33] Hänzelmann S Castelo R Guinney J . GSVA: gene set variation analysis for microarray and RNA-seq data. BMC Bioinf. (2013) 14:7. doi: 10.1186/1471-2105-14-7, PMID: 23323831 PMC3618321

[34] Maeser D Gruener RF Huang RS . oncoPredict: an R package for predicting *in vivo* or cancer patient drug response and biomarkers from cell line screening data. Brief Bioinform. (2021) 22. doi: 10.1093/bib/bbab260, PMID: 34260682 PMC8574972

[35] Cable DM Murray E Zou LS Goeva A Macosko EZ Chen F . Robust decomposition of cell type mixtures in spatial transcriptomics. Nat Biotechnol. (2022) 40:517–26. doi: 10.1038/s41587-021-00830-w, PMID: 33603203 PMC8606190

[36] Tanevski J Flores ROR Gabor A Schapiro D Saez-Rodriguez J . Explainable multiview framework for dissecting spatial relationships from highly multiplexed data. Genome Biol. (2022) 23:97. doi: 10.1186/s13059-022-02663-5, PMID: 35422018 PMC9011939

[37] Hou Y Wang S Zhang Y Huang X Zhang X He F . Proteomics identifies LUC7L3 as a prognostic biomarker for hepatocellular carcinoma. Curr Issues Mol Biol. (2024) 46:4004–20. doi: 10.3390/cimb46050247, PMID: 38785515 PMC11120364

[38] Shen H Gu X Li H Tang M Li X Zhang Y . Exploring prognosis, tumor microenvironment and tumor immune infiltration in hepatocellular carcinoma based on ATF/CREB transcription factor family gene-related model. J Hepatocell Carcinoma. (2023) 10:327–45. doi: 10.2147/jhc.S398713, PMID: 36874250 PMC9983578

[39] Wang Y Deng B . Hepatocellular carcinoma: molecular mechanism, targeted therapy, and biomarkers. Cancer metastasis Rev. (2023) 42:629–52. doi: 10.1007/s10555-023-10084-4, PMID: 36729264

[40] Ayuso C Rimola J Vilana R Burrel M Darnell A García-Criado Á . Diagnosis and staging of hepatocellular carcinoma (HCC): current guidelines. Eur J radiology. (2018) 101:72–81. doi: 10.1016/j.ejrad.2018.01.025, PMID: 29571804

[41] Vilgrain V . Advancement in HCC imaging: diagnosis, staging and treatment efficacy assessments: hepatocellular carcinoma: imaging in assessing treatment efficacy. J hepato-biliary-pancreatic Sci. (2010) 17:374–9. doi: 10.1007/s00534-009-0230-3, PMID: 19924373

[42] Yang C Zhang H Zhang L Zhu AX Bernards R Qin W . Evolving therapeutic landscape of advanced hepatocellular carcinoma. Nat Rev Gastroenterol hepatology. (2023) 20:203–22. doi: 10.1038/s41575-022-00704-9, PMID: 36369487

[43] Llovet JM De Baere T Kulik L Haber PK Greten TF Meyer T . Locoregional therapies in the era of molecular and immune treatments for hepatocellular carcinoma. Nat Rev Gastroenterol hepatology. (2021) 18:293–313. doi: 10.1038/s41575-020-00395-0, PMID: 33510460

[44] Wang J Sun L Liu Y Zhang Y . FIGNL1 promotes hepatocellular carcinoma formation via remodeling ECM-receptor interaction pathway mediated by HMMR. Curr Gene Ther. (2024) 24:249–63. doi: 10.2174/0115665232274223231017052707, PMID: 37929733 PMC11071652

[45] Liu C Shi J Lin B Zhou M Shan D Nie J . SHR6390 combined with cabozantinib inhibits tumor progression in the hepatocellular carcinoma mouse model. Curr Gene Ther. (2024) 24:453–64. doi: 10.2174/1566523222666220825110147, PMID: 36017825

[46] Hernandez-Gea V Friedman SL . Pathogenesis of liver fibrosis. Annu Rev pathology. (2011) 6:425–56. doi: 10.1146/annurev-pathol-011110-130246, PMID: 21073339

[47] Dhar D Baglieri J Kisseleva T Brenner DA . Mechanisms of liver fibrosis and its role in liver cancer. Exp Biol Med (Maywood NJ). (2020) 245:96–108. doi: 10.1177/1535370219898141, PMID: 31924111 PMC7016420

[48] Pinter M Scheiner B Pinato DJ . Immune checkpoint inhibitors in hepatocellular carcinoma: emerging challenges in clinical practice. Lancet Gastroenterol hepatology. (2023) 8:760–70. doi: 10.1016/s2468-1253(23)00147-4, PMID: 37327807

[49] Yu Z Wu X Zhu J Yan H Li Y Zhang H . BCLAF1 binds SPOP to stabilize PD-L1 and promotes the development and immune escape of hepatocellular carcinoma. Cell Mol Life sciences: CMLS. (2024) 81:82. doi: 10.1007/s00018-024-05144-z, PMID: 38340178 PMC10858942

[50] Dhanasekaran R Hansen AS Park J Lemaitre L Lai I Adeniji N . MYC overexpression drives immune evasion in hepatocellular carcinoma that is reversible through restoration of proinflammatory macrophages. Cancer Res. (2023) 83:626–40. doi: 10.1158/0008-5472.Can-22-0232, PMID: 36525476 PMC9931653

[51] Qi L Tan Y Zhou Y Dong Y Yang X Chang S . Proteogenomic identification and analysis of KIF5B as a prognostic signature for hepatocellular carcinoma. Curr Gene Ther. (2025) 25:532–45. doi: 10.2174/0115665232308821240826075513, PMID: 39248070

[52] Xue C Gu X Zheng Q Shi Q Yuan X Chu Q . Effects of 3-HAA on HCC by regulating the heterogeneous macrophages-A scRNA-seq analysis. Adv Sci (Weinh). (2023) 10:e2207074. doi: 10.1002/advs.202207074, PMID: 37013458 PMC10238176

[53] Shi Q Chu Q Zeng Y Yuan X Wang J Zhang Y . Non-coding RNA methylation modifications in hepatocellular carcinoma: interactions and potential implications. Cell Commun Signal. (2023) 21:359. doi: 10.1186/s12964-023-01357-0, PMID: 38111040 PMC10726651

[54] Li Q Han J Yang Y Chen Y . PD-1/PD-L1 checkpoint inhibitors in advanced hepatocellular carcinoma immunotherapy. Front Immunol. (2022) 13:1070961. doi: 10.3389/fimmu.2022.1070961, PMID: 36601120 PMC9806143

[55] Gao Y You M Fu J Tian M Zhong X Du C . Intratumoral stem-like CCR4+ regulatory T cells orchestrate the immunosuppressive microenvironment in HCC associated with hepatitis B. J hepatology. (2022) 76:148–59. doi: 10.1016/j.jhep.2021.08.029, PMID: 34689996

[56] Ruf B Bruhns M Babaei S Kedei N Ma L Revsine M . Tumor-associated macrophages trigger MAIT cell dysfunction at the HCC invasive margin. Cell. (2023) 186:3686–3705.e32. doi: 10.1016/j.cell.2023.07.026, PMID: 37595566 PMC10461130

[57] Ye W Wang J Zheng J Jiang M Zhou Y Wu Z . Association between higher expression of Vav1 in hepatocellular carcinoma and unfavourable clinicopathological features and prognosis. Protein Pept Lett. (2024) 31:706–13. doi: 10.2174/0109298665330781240830042601, PMID: 39301900

[58] He X Ma J Yan X Yang X Wang P Zhang L . CDT1 is a potential therapeutic target for the progression of NAFLD to HCC and the exacerbation of cancer. Curr Genomics. (2025) 26:225–43. doi: 10.2174/0113892029313473240919105819, PMID: 40433415 PMC12107793

[59] Xiong YM Zhou F Zhou JW Liu F Zhou SQ Li B . Aberrant expressions of PSMD14 in tumor tissue are the potential prognostic biomarkers for hepatocellular carcinoma after curative resection. Curr Genomics. (2023) 24:368–84. doi: 10.2174/0113892029277262231108105441, PMID: 38327651 PMC10845065

[60] Mu R Chang M Feng C Cui Y Li T Liu C . Analysis of the expression of PRDX6 in patients with hepatocellular carcinoma and its effect on the phenotype of hepatocellular carcinoma cells. Curr Genomics. (2024) 25:2–11. doi: 10.2174/0113892029273682240111052317, PMID: 38544826 PMC10964084

[61] Zhou Y Gu J Yu H Chen F Long C Maihemuti M . Screening and identification of ESR1 as a target of icaritin in hepatocellular carcinoma: evidence from bibliometrics and bioinformatic analysis. Curr Mol Pharmacol. (2024) 17:e18761429260902. doi: 10.2174/0118761429260902230925044009, PMID: 38239068

[62] Guo J Jin G Hu Y Zhao Z Nan F Hu X . Wogonin restrains the Malignant progression of lung cancer through modulating MMP1 and PI3K/AKT signaling pathway. Protein Pept Lett. (2023) 30:25–34. doi: 10.2174/0929866530666221027152204, PMID: 36305146

[63] Liu Z Zhang J Liu J Guo L Chen G Fang Y . Combining network pharmacology, molecular docking and preliminary experiments to explore the mechanism of action of FZKA formula on non-small cell lung cancer. Protein Pept Lett. (2023) 30:1038–47. doi: 10.2174/0109298665268153231024111622, PMID: 37962044

[64] Du Y Shi J Wang J Xun Z Yu Z Sun H . Integration of pan-cancer single-cell and spatial transcriptomics reveals stromal cell features and therapeutic targets in tumor microenvironment. Cancer Res. (2024) 84:192–210. doi: 10.1158/0008-5472.Can-23-1418, PMID: 38225927

[65] Halpern KB Shenhav R Massalha H Toth B Egozi A Massasa EE . Paired-cell sequencing enables spatial gene expression mapping of liver endothelial cells. Nat Biotechnol. (2018) 36:962–70. doi: 10.1038/nbt.4231, PMID: 30222169 PMC6546596

[66] Lai H Cheng X Liu Q Luo W Liu M Zhang M . Single-cell RNA sequencing reveals the epithelial cell heterogeneity and invasive subpopulation in human bladder cancer. Int J cancer. (2021) 149:2099–115. doi: 10.1002/ijc.33794, PMID: 34480339

[67] Robles-Remacho A Sanchez-Martin RM Diaz-Mochon JJ . Spatial transcriptomics: emerging technologies in tissue gene expression profiling. Analytical Chem. (2023) 95:15450–60. doi: 10.1021/acs.analchem.3c02029, PMID: 37814884 PMC10603609

[68] Massalha H Bahar Halpern K Abu-Gazala S Jana T Massasa EE Moor AE . A single cell atlas of the human liver tumor microenvironment. Mol Syst Biol. (2020) 16:e9682. doi: 10.15252/msb.20209682, PMID: 33332768 PMC7746227

[69] Zhang L Chen D Song D Liu X Zhang Y Xu X . Clinical and translational values of spatial transcriptomics. Signal transduction targeted Ther. (2022) 7:111. doi: 10.1038/s41392-022-00960-w, PMID: 35365599 PMC8972902

[70] Huang X Liu J Mo X Liu H Wei C Huang L . Systematic profiling of alternative splicing events and splicing factors in left- and right-sided colon cancer. Aging. (2019) 11:8270–93. doi: 10.18632/aging.102319, PMID: 31586988 PMC6814588

[71] Li Y Fu Y Hu X Sun L Tang D Li N . The HBx-CTTN interaction promotes cell proliferation and migration of hepatocellular carcinoma via CREB1. Cell Death disease. (2019) 10:405. doi: 10.1038/s41419-019-1650-x, PMID: 31138777 PMC6538608

[72] Pushpamithran G Blomgran R . Macrophage-derived extracellular vesicles from Ascaris lumbricoides antigen exposure enhance Mycobacterium tuberculosis growth control, reduce IL-1β, and contain miR-342-5p, miR-516b-5p, and miR-570-3p that regulate PI3K/AKT and MAPK signaling pathways. Front Immunol. (2024) 15:1454881. doi: 10.3389/fimmu.2024.1454881, PMID: 39569198 PMC11576181

[73] Kranjc T Dempsey E Cagney G Nakamura N Shields DC Simpson JC . Functional characterisation of the YIPF protein family in mammalian cells. Histochem Cell Biol. (2017) 147:439–51. doi: 10.1007/s00418-016-1527-3, PMID: 27999994

[74] Müller M Wasson CW Bhatia R Boxall S Millan D Goh GY . YIP1 family member 4 (YIPF4) is a novel cellular binding partner of the papillomavirus E5 proteins. Sci Rep. (2015) 5:12523. doi: 10.1038/srep12523, PMID: 26235900 PMC4522686

