# Supplementary figures and images for: Integrating single-cell RNA sequencing with spatial transcriptomics reveal the fibrosis-related genes in hepatocellular carcinoma
Source: Front Immunol. 2026 Jan 14;16:1659404. doi: 10.3389/fimmu.2025.1659404 (PMC12847382; doi:10.3389/fimmu.2025.1659404)

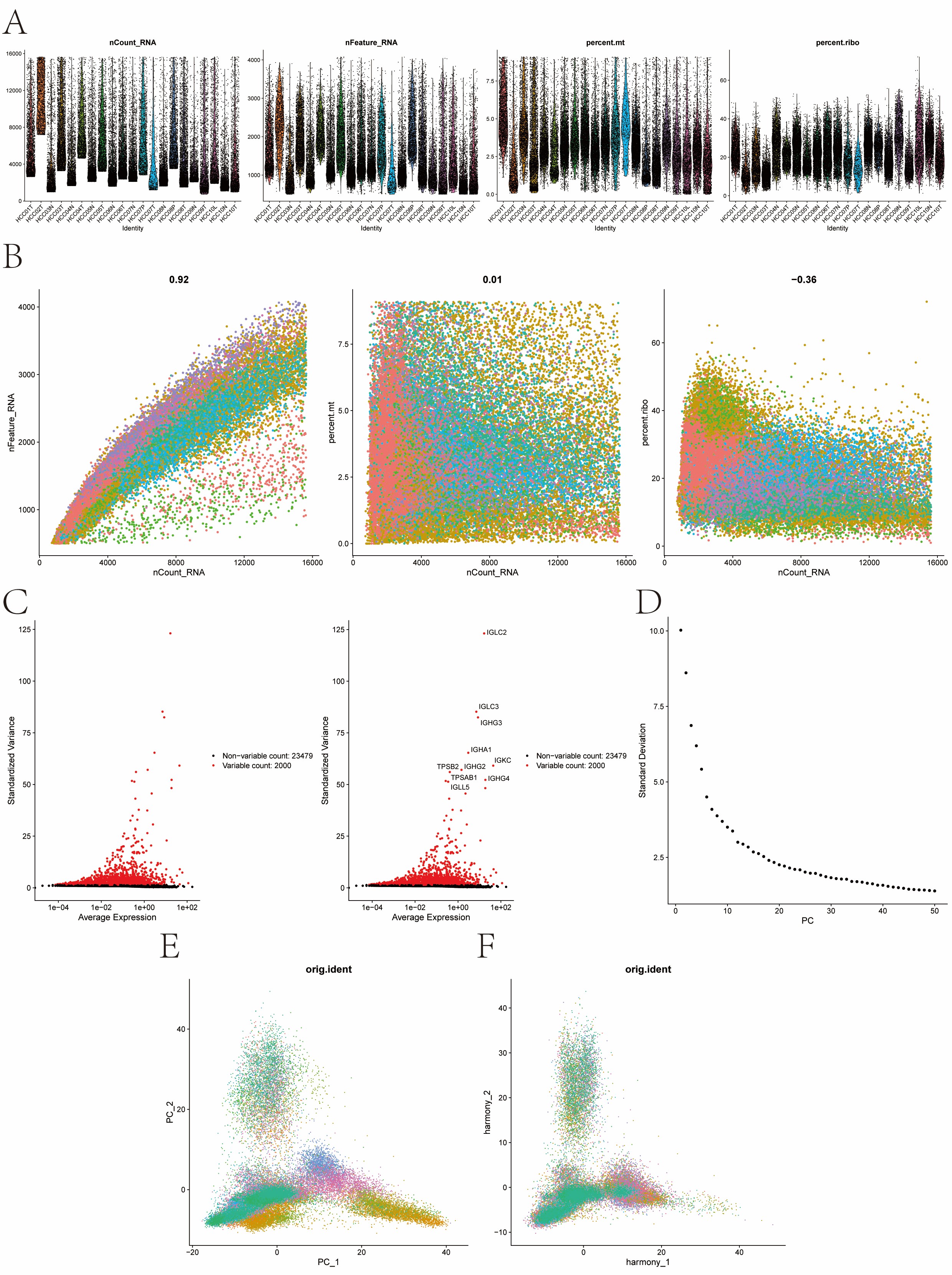

Supplement: Supplementary Figure 1 — The quality control before and after filtration. [file Image1.jpeg]

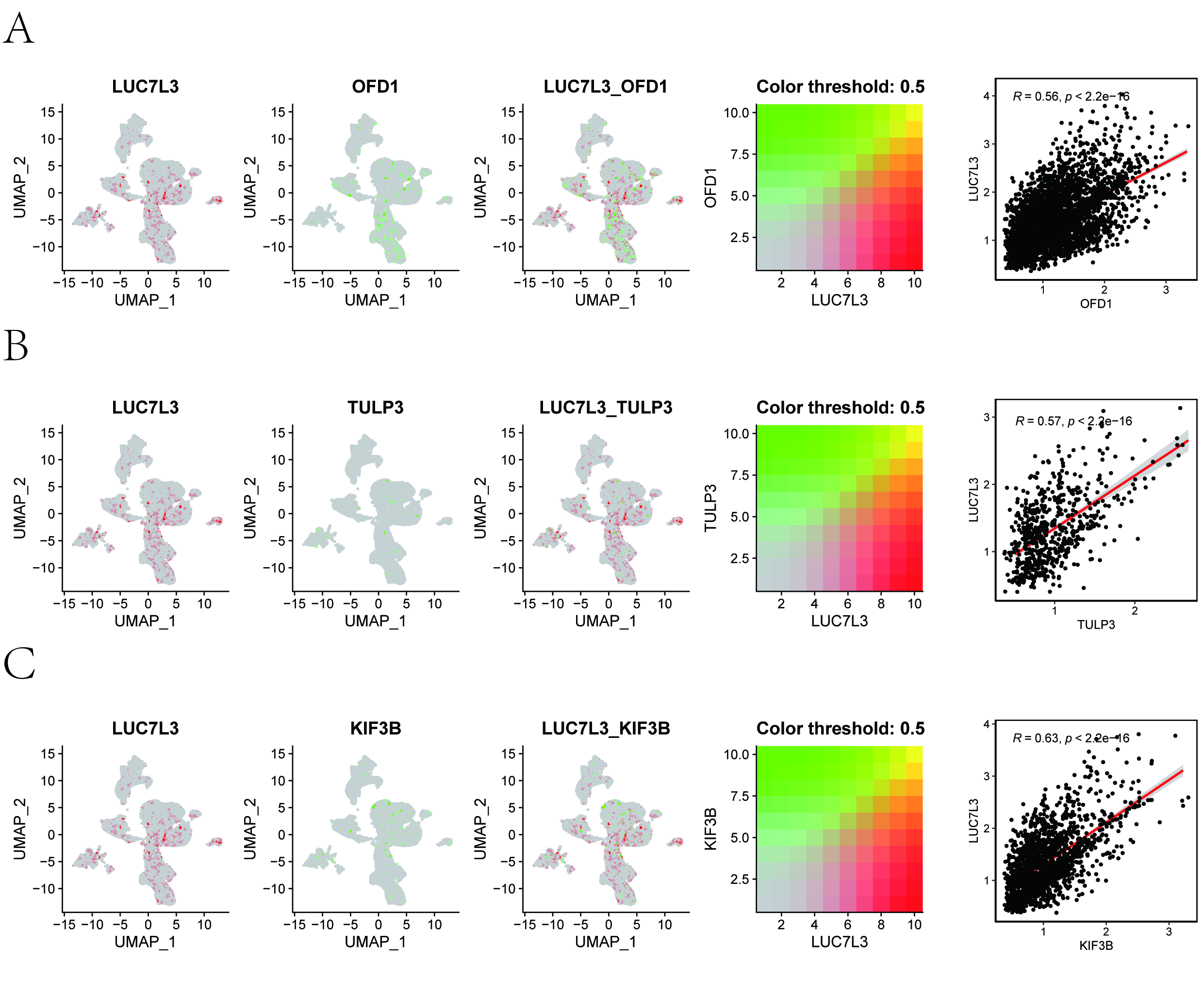

Supplement: Supplementary Figure 2 — Co-expression analysis between fibrosis-related genes and LUC7L3. (A) Co-expression analysis between OFD1 and LUC7L3. (B) Co-expression analysis between TULP3 and LUC7L3. (C) Co-expression analysis between KIF3B and LUC7L3. [file Image2.tif]

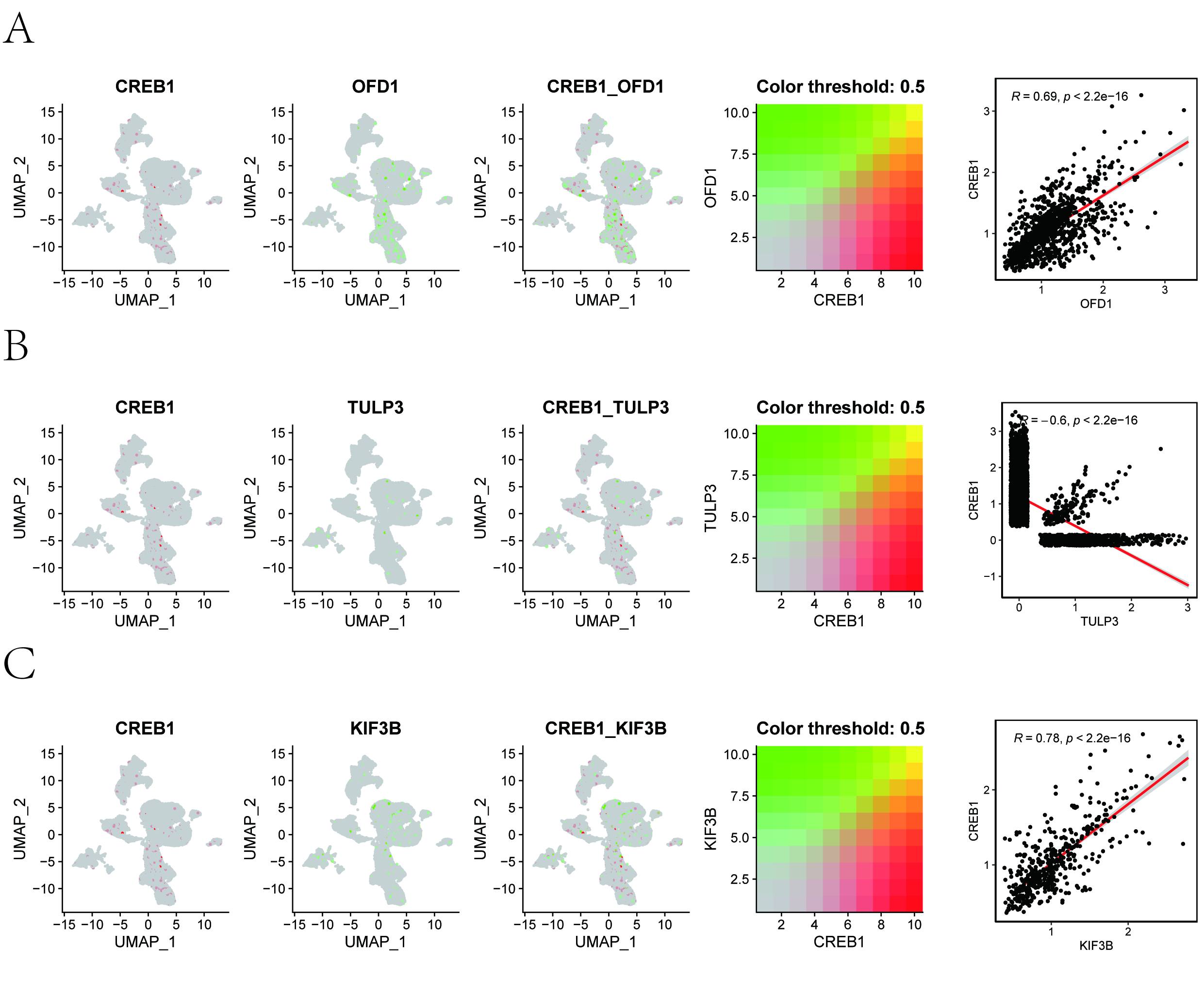

Supplement: Supplementary Figure 3 — Co-expression analysis between fibrosis-related genes and CREB1. (A) Co-expression analysis between OFD1 and CREB1. (B) Co-expression analysis between TULP3 and CREB1. (C) Co-expression analysis between KIF3B and CREB1. [file Image3.tif]

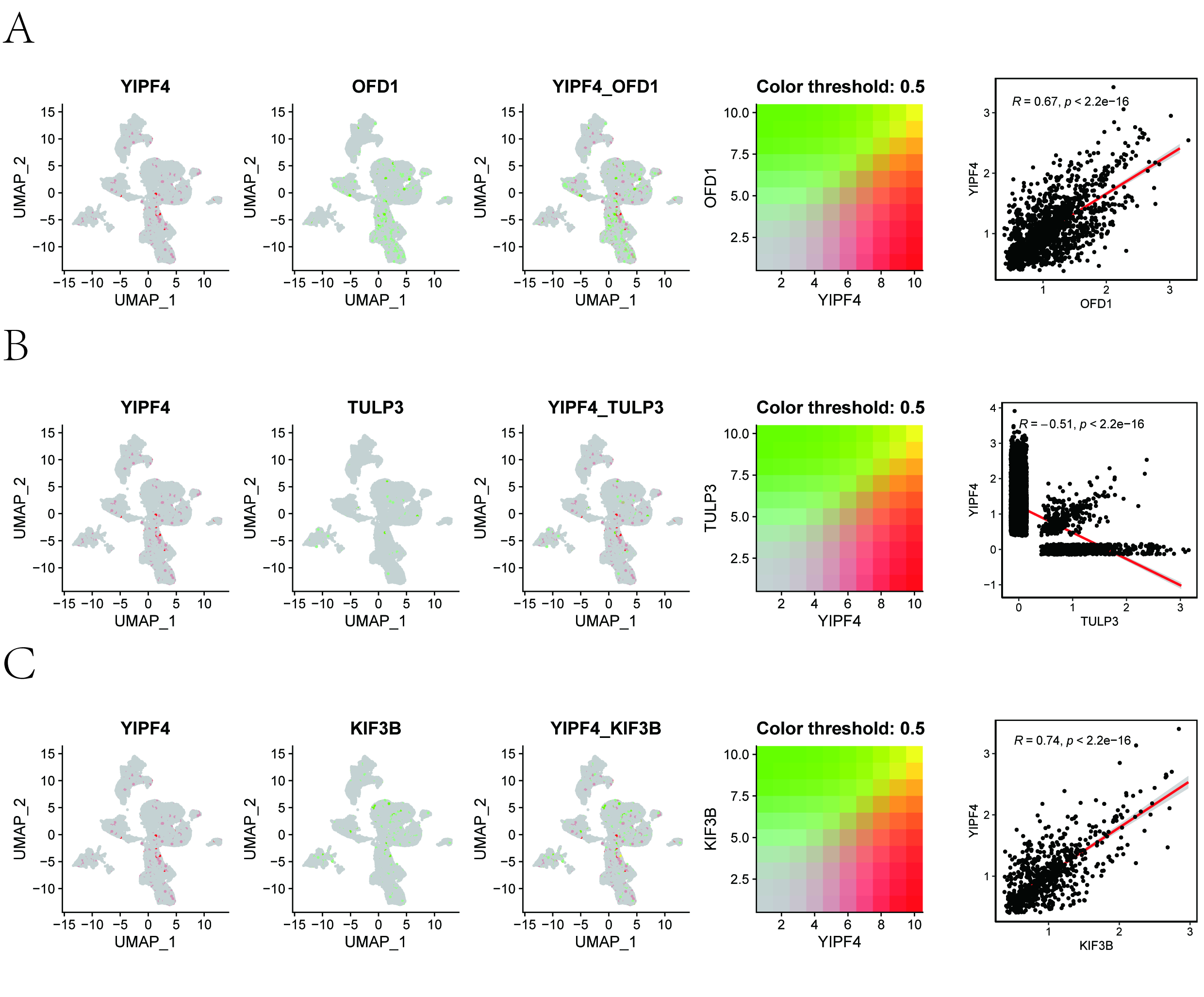

Supplement: Supplementary Figure 4 — Co-expression analysis between fibrosis-related genes and YIPF4. (A) Co-expression analysis between OFD1 and YIPF4. (B) Co-expression analysis between TULP3 and YIPF4. (C) Co-expression analysis between KIF3B and YIPF4. [file Image4.tif]

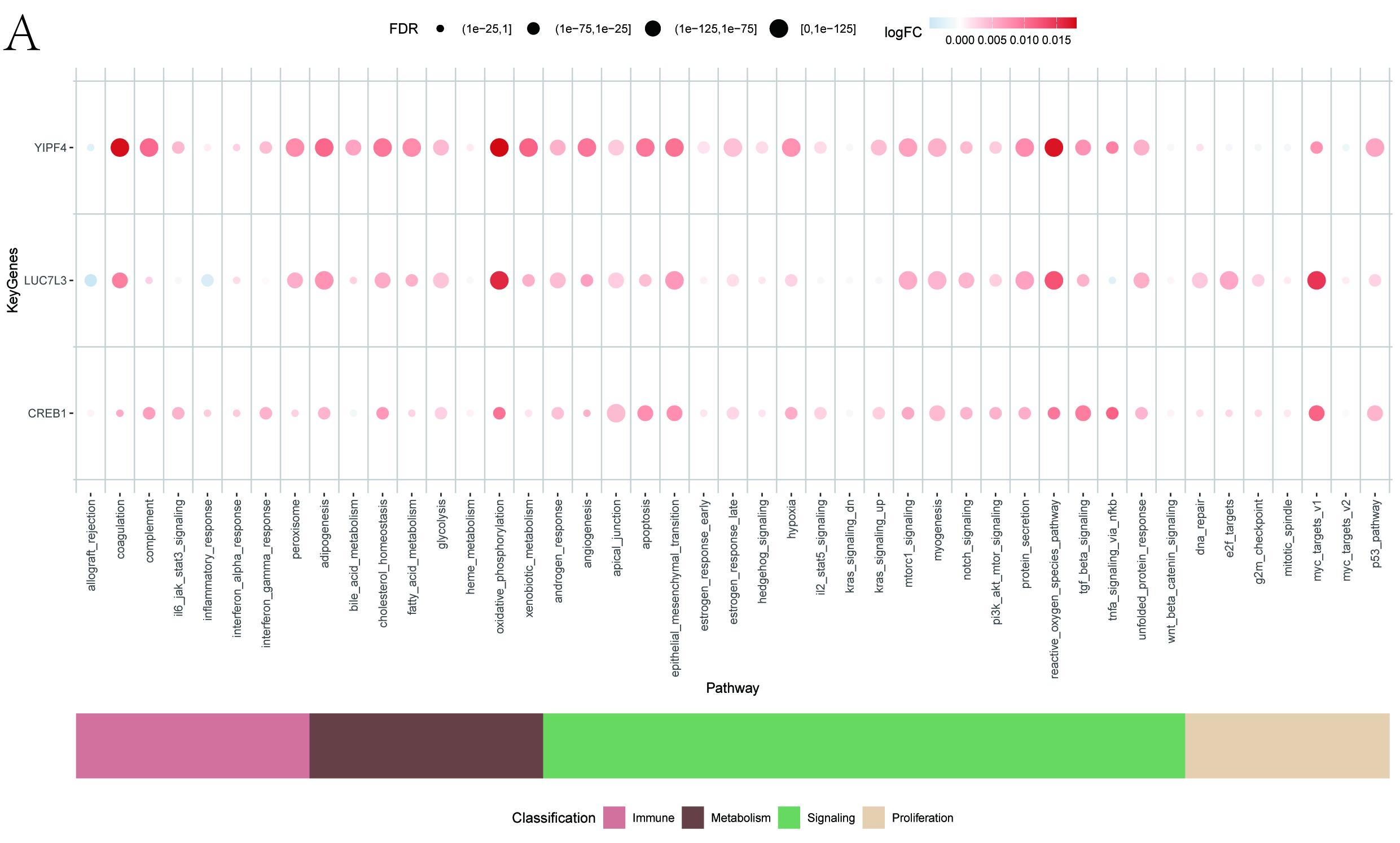

Supplement: Supplementary Figure 5 — Immunometabolism-related pathways for three key genes. [file Image5.tif]

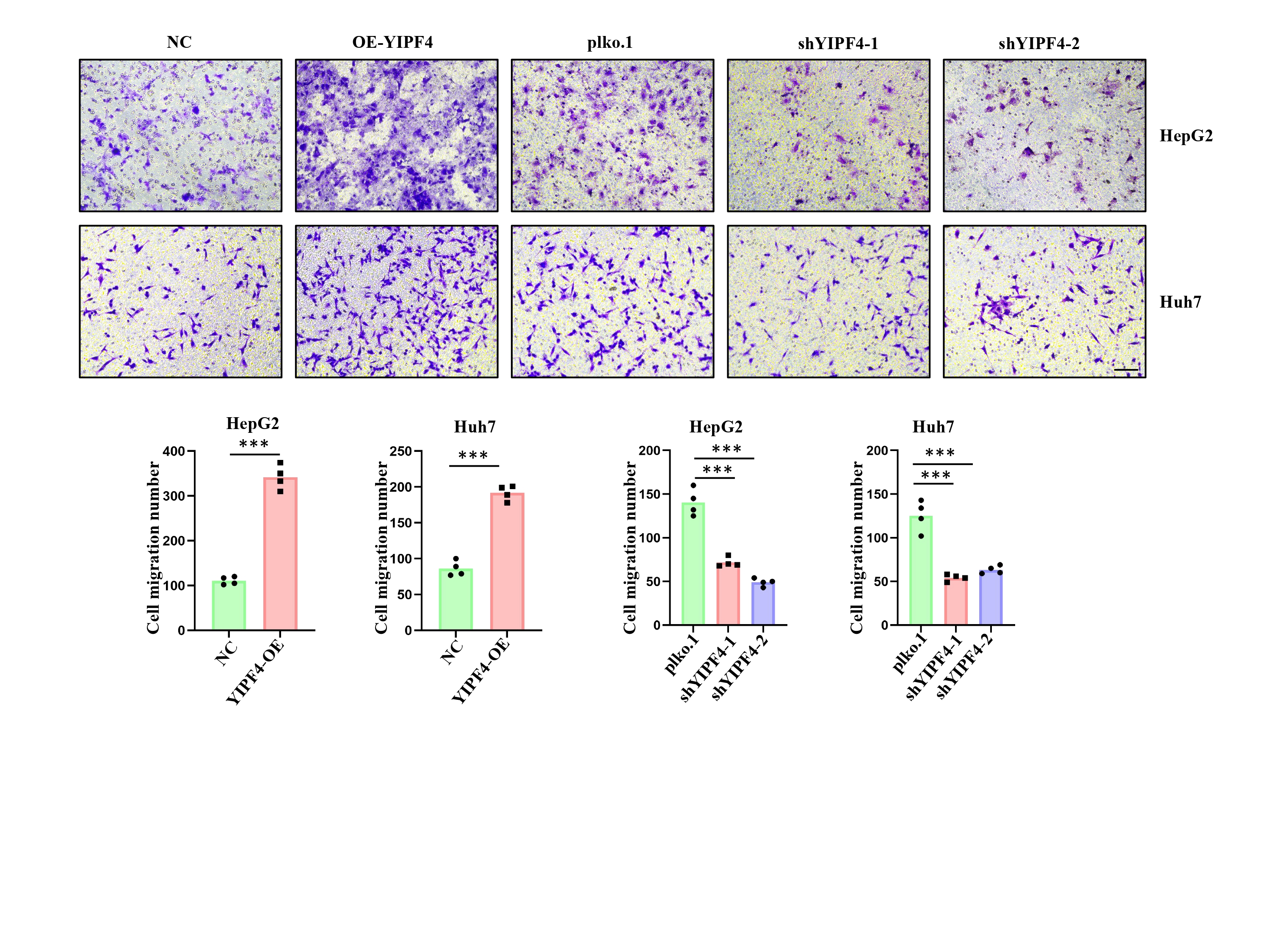

Supplement: Supplementary Figure 6 — Transwell migration assay for YIPF4 in HCC cell lines. [file Image6.jpeg]

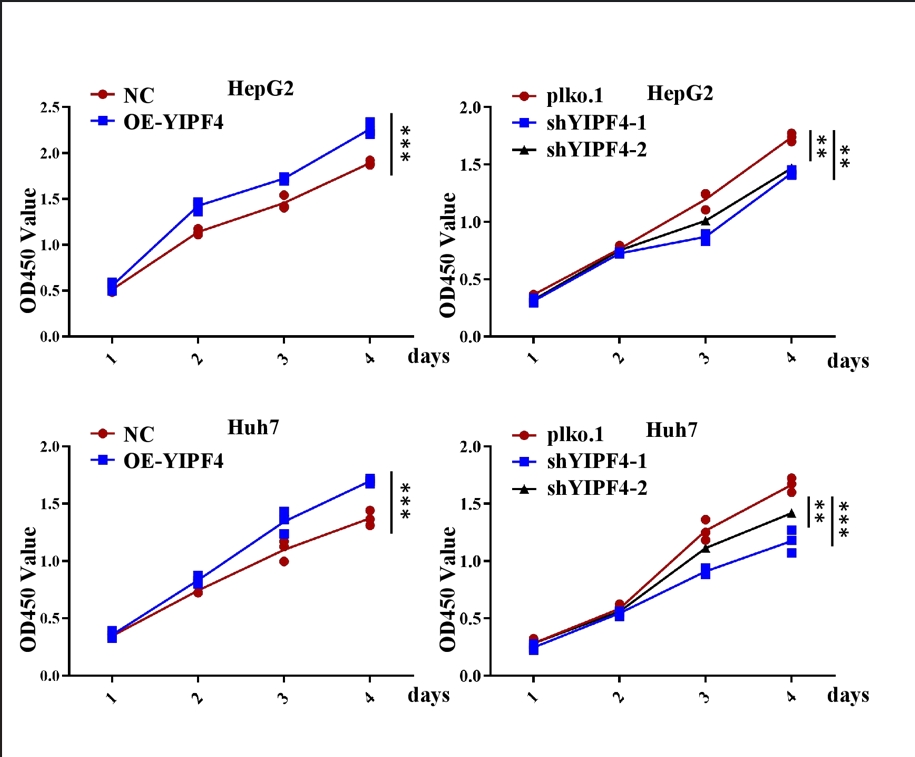

Supplement: Supplementary Figure 7 — CCK-8 proliferation assay for YIPF4 in HCC cell lines. [file Image7.jpeg]
